# Energy Exchanges in Saturn's Polar Regions From Cassini Observations: Eddy‐Zonal Flow Interactions

**DOI:** 10.1029/2021JE006973

**Published:** 2022-04-27

**Authors:** Peter L. Read, Arrate Antuñano, Simon Cabanes, Greg Colyer, Teresa del Río Gaztelurrutia, Agustin Sanchez‐Lavega

**Affiliations:** ^1^ Atmospheric, Oceanic and Planetary Physics Department of Physics University of Oxford Clarendon Laboratory Oxford UK; ^2^ School of Physics and Astronomy University of Leicester University Road Leicester UK; ^3^ Dpto de Física Aplicada, Escuela de Ingeniería de Bilbao UPV/EHU Bilbao Spain; ^4^ DICEA Sapienza Università di Roma Rome Italy

**Keywords:** Saturn, atmospheric dynamics, energetics, zonal jets, polar vortices

## Abstract

Saturn's polar regions (polewards of ∼63° planetocentric latitude) are strongly dynamically active with zonal jets, polar cyclones and the intriguing north polar hexagon (NPH) wave. Here we analyze measurements of horizontal winds, previously obtained from Cassini images by Antuñano et al. (2015), https://doi.org/10.1002/2014je004709, to determine the spatial and spectral exchanges of kinetic energy (KE) between zonal mean zonal jets and nonaxisymmetric eddies in Saturn's polar regions. Eddies of most resolved scales generally feed KE into the eastward and westward zonal mean jets at rates between 4.3 × 10^−5^ and 1.4 × 10^−4^ W kg^−1^. In particular, the north polar jet (at 76°N) was being energized at a rate of ∼10^−4^ W kg^−1^, dominated by the contribution due to the zonal wavenumber *m* = 6 NPH wave itself. This implies that the hexagon was not being driven at this time through a barotropic instability of the north polar jet, but may suggest a significant role for baroclinic instabilities, convection or other internal energy sources for this feature. The south polar zonal mean jet KE was also being sustained by eddies in that latitude band across a wide range of *m*. In contrast, results indicate that the north polar vortex may have been weakly barotropically unstable at this time with eddies of low *m* gaining KE at the expense of the axisymmetric cyclone. However, the southern axisymmetric polar cyclone was gaining KE from non‐axisymmetric components at this time, including *m* = 2 and its harmonics, as the elliptical distortion of the vortex may have been decaying.

## Key Points

- Wind velocities obtained from Cassini images are analyzed to determine eddy‐zonal mean exchanges of kinetic energy (KE) in Saturn's polar regions
- Both the North and South Polar zonal mean jets at 76^°^N and 70^°^S are energized from non‐zonal eddies, including the *m* = 6 North Polar Hexagon wave
- The North Polar Vortex was barotropically unstable at this time, but the South Polar Vortex was gaining net KE from eddies

## 1 Introduction

**Figure 1:**
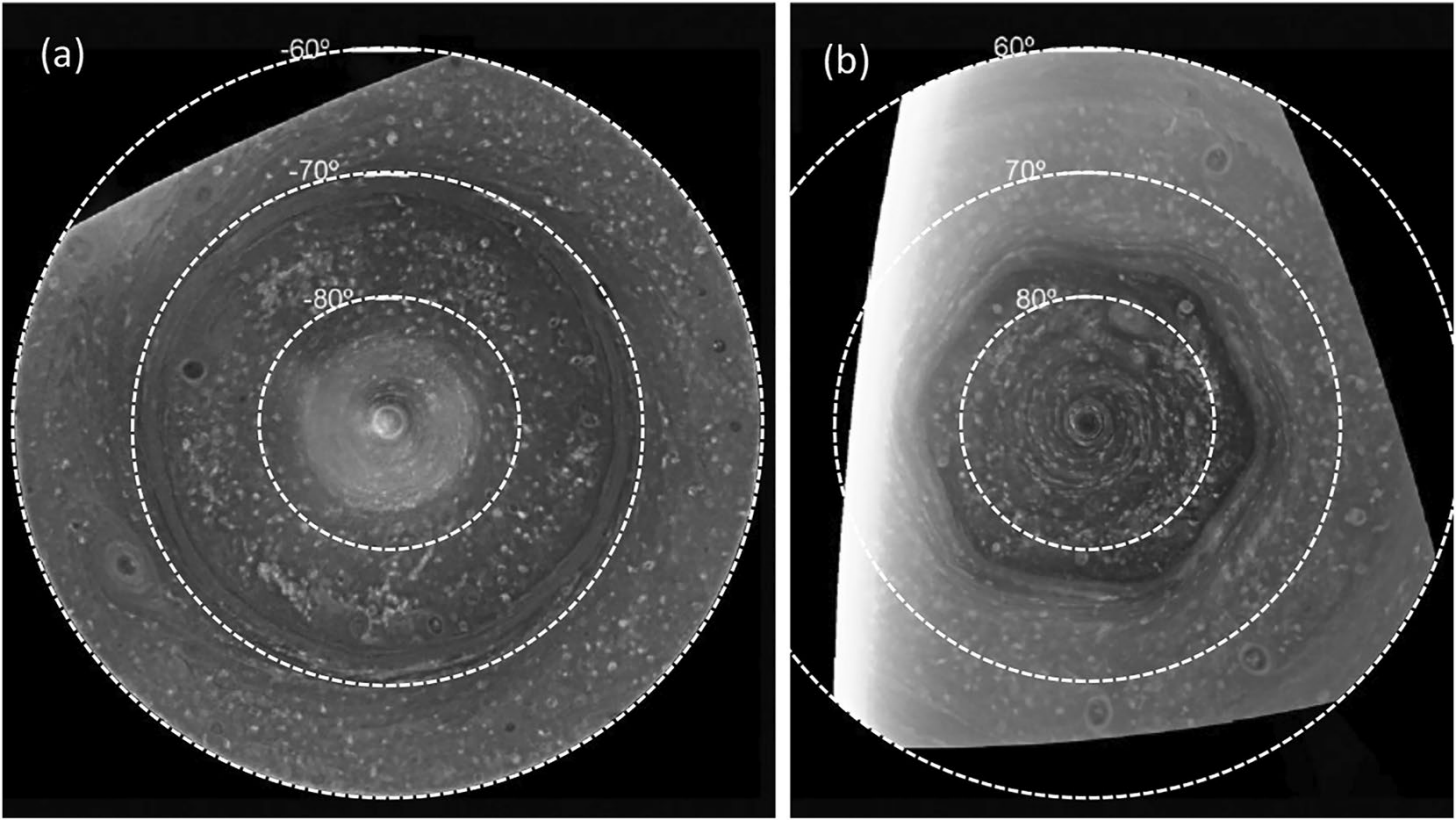
(a) Polar projection of the south polar region from 60° to 90°S, built using four different images captured by the Cassini Imaging Sub‐System wide‐angle camera with a CB2 filter on 3 December 2008. (b) An equivalent projection of the north polar region from 60° to 90°S, also from a Cassini wide‐angle camera image obtained with a CB2 filter on 14 June 2013 (adapted from Antuñano et al. (2015), their Figure 1, with permission).

Since the Cassini orbiter mission to Saturn, it has been clear (Antuñano et al., 2015; Baines et al., 2009; Dyudina et al., 2008, 2009; Sánchez‐Lavega et al., 2006; Sayanagi et al., 2017, 2018) that its polar regions are dominated at the cloud‐top levels by intense, cyclonic vortices, centered on each pole, surrounded by an additional eastward jet stream at latitude 70°S and 76°N respectively (planetocentric); see Figure 1. The polar vortices in both hemispheres extend to a radius of around 5° colatitude, corresponding to around 4,700 km (Liu et al., 2019; Sánchez‐Lavega et al., 2006; Sayanagi et al., 2017), with strong circumpolar jets peaking at around 87° latitude with velocities of up to 160–175 m s^−1^. The vortices appear to be roughly circular, with spiral cloud bands and an apparent clearing at the center of each vortex, reminiscent of terrestrial tropical cyclones. But high resolution images (Baines et al., 2009; Dyudina et al., 2008, 2009; Liu et al., 2019; Sánchez‐Lavega et al., 2006; Sayanagi et al., 2017) indicate many small‐scale cloudy features that break the circular symmetry.

Weak westward zonal flow is found immediately beyond the edge of each polar vortex, reversing at lower latitudes to form the secondary eastward circumpolar jets in the zonal mean (South Polar Jet and North Polar Jet; SPJ and NPJ) at approximately 70° and 76° planetocentric latitude in the southern and northern hemispheres respectively (note that all latitudes in this paper are planetocentric) before reversing again at even lower latitudes. The North Polar Jet (NPJ) is notable for its regular hexagonal shape, first discovered in Voyager images by Godfrey (1988). This North Polar Hexagon (NPH) feature has evidently persisted to the present day, and was observed in detail by the Cassini orbiter (e.g., Baines et al., 2009; Fletcher et al., 2018) in both cloud motions and in the retrievals of temperature in the lower stratosphere from Cassini Composite InfraRed Spectrometer measurements. Such a polygonal perturbation to the jet is not seen in the South Polar Jet (SPJ) (Sánchez‐Lavega et al., 2002), however, for reasons that are still poorly understood.

Indeed the nature and origin of both the polar cyclones and the North Polar Hexagon (NPH) meanders continues to pose major challenges to atmospheric scientists (see Sayanagi et al., 2018, for a recent review), prompting a continuing need for more observational information with which to constrain theories and models. The resemblance of the polar vortices to terrestrial hurricanes, for example, would suggest a need for localized heating for example, produced by latent heat release in moist convection (e.g., O’Neill et al., 2016, 2015; Sayanagi et al., 2017). But the compact morphology of terrestrial tropical cyclones is due in part to concentrated convergence and upwelling in the atmosphere associated with the underlying ocean surface (e.g., Montgomery & Smith, 2017), which is likely absent on Saturn.

The North Polar Hexagon (NPH) has been the subject of much discussion since its discovery, not least because of its remarkable symmetry and stable persistence over several decades. Initial studies noted a possible association between the hexagon wave and a large anticyclonic vortex, known as the North Polar Spot (NPS), lying just outside the main jet at the time of the Voyager encounters (Godfrey, 1988), suggesting that the anticyclone was perturbing the circumpolar jet to induce a train of Rossby waves with a wavelength just matching the wavenumber *m* = 6 pattern at this latitude (Allison et al., 1990; Sánchez‐Lavega et al., 1997). Subsequent observations from the Hubble Space Telescope showed that the North Polar Spot (NPS) persisted into the 1990s, but by the time the Cassini Orbiter arrived at Saturn it had disappeared. Cassini observations, however, showed that the NPH was still present even without the presence of the North Polar Spot (NPS), implying that the hexagon wave was not being maintained by the NPS.

More recent explanations proposed for the origin of the NPH attribute it either to a Rossby wave propagating upwards from a (nearly stationary) source in the deep interior (Sánchez‐Lavega et al., 2014) or to an equilibrated instability (barotropic or baroclinic) of either a relatively shallow, initially axisymmetric North Polar Jet (NPJ) itself (e.g., Aguiar et al., 2010; Farrell & Ioannou, 2017; Morales‐Juberías et al., 2015, 2011; Rostami et al., 2017) or deep jets driven by deep planetary convection (Garcia et al., 2020; Yadav & Bloxham, 2020). The formation of polygonal jet flows as the fully developed form of either barotropic or baroclinic instabilities is well known in laboratory experiments (e.g., Aguiar et al., 2010; Bastin & Read, 1998; Früh & Read, 1999; Hide & Mason, 1975; Sommeria et al., 1991, 1989) though are much less commonly found in planetary atmospheres (however, cf. Yadav & Bloxham, 2020). Equilibrated barotropic instabilities of plausible zonal jets were commonly found to be associated with chains of cyclonic or anticyclonic vortices alternately inside and outside of the meandering jet (Aguiar et al., 2010; Morales‐Juberías et al., 2011; Yadav & Bloxham, 2020). Such vortex chains are not observed prominently on Saturn (Antuñano et al., 2015), though such features could conceivably be very weak or imperceptibile in some model parameter regimes with more complex vertical structure (e.g., Morales‐Juberías et al., 2015). Baroclinic instabilities in stably‐stratified flows may also lead to equilibrated meandering polygonal jet structures at certain levels in the vertical, with or without accompanying vortices (e.g., Bastin & Read, 1997, 1998). Such regimes may persist for as long as the initial jet is maintained. A plausible complete solution, for example, in which a jet is sustained by upscale kinetic energy (KE) transfers from small‐scale eddies and develops a large‐scale polygonal, meandering, wave‐like barotropic instability, has been demonstrated in a two‐layer numerical simulation by Farrell and Ioannou (2017). Such a “flux loop” mechanism emulates aspects of a similar scenario in two‐dimensional stratified turbulence identified by Boffetta et al. (2011).

Observations have indicated that the maintenance of alternating jet flows on Saturn, at least at extra‐tropical middle latitudes, is associated with strongly divergent or convergent Reynolds stresses that directly accelerate the zonal flow (Del Genio & Barbara, 2012; Del Genio et al., 2007) in a spectrally non‐local transfer (i.e., direct from non‐axisymmetric to zonal flow rather than via an incremental cascade) of kinetic energy (KE). This is similar to what has been found at mid‐low latitudes in Jupiter's atmosphere, with an inferred mean transfer rate of ∼10^−5^ – 10^−4^ W kg^−1^ (Ingersoll et al., 1981; Salyk et al., 2006; Sromovsky et al., 1982). The sign and magnitude of the conversion rate of eddy kinetic energy (EKE) at latitudes higher than ±60° has not so far been determined (for either planet). Similarly, exchanges of KE between the NPH wave and other components of the flow have yet to be determined. Yet such statistics may shed important light on the nature of the NPH and other features at these high latitudes and provide important constraints on plausible models of these phenomena.

In the present work, therefore, we extend the analysis of the velocity field measurements of Antuñano et al. (2015) to explore the zonal KE spectra of both polar regions of Saturn and estimate the sign and magnitude of the rates of exchange of KE between the zonal mean jet flows and non‐axisymmetric components of the flow (hereafter referred to as “eddies”). The data prove sufficient to obtain robust estimates of the total eddy‐zonal mean conversion rate of kinetic energy for both polar regions and more locally in the vicinity of the South Polar Jet (SPJ), NPJ and both polar vortices. A zonal spectral decomposition of this conversion rate also allows a determination of the interaction between the *m* = 6 NPH meanders and the zonal mean NPJ and other features.

Section 2 summarizes the observations used and the methods applied to obtain the kinetic energy spectra. Section 3 describes the methods used to compute the eddy‐zonal KE conversion rates and spectral and spatial fluxes. Section 4 presents the results on the eddy‐zonal flow energy exchanges, including regional variations and their spectral decomposition. The results and their significance are discussed in Section 5 together with conclusions and suggestions for further work.

## 2 Observations

The observations used in the present study consist of two maps of horizontal velocities in Saturn's northern and southern hemispheres, as previously published by Antuñano et al. (2015). As fully described in that paper, these measurements were derived from sets of Cassini Imaging Sub‐System (ISS) Wide Angle Camera (WAC) and Narrow Angle Camera (NAC) images using the continuum band CB2 and CB3 filters, acquired for the northern hemisphere in June 2013 and for the southern hemisphere using Wide Angle Camera (WAC) CB2 and CB3 images taken in October 2006 and December 2008. Additional Narrow Angle Camera (NAC) images using the CB2 and red filters taken in July 2008 were also used to analyze the southern polar vortex. The Wide Angle Camera (WAC) images covered a region extending from a planetocentric latitude of around 60°–65° to each pole (apart from a segment in longitude between around 35°–110°W in the south) with a horizontal resolution equivalent to around 0.05° latitude (around 50 km) per pixel, while Narrow Angle Camera (NAC) images were mostly used for the polar vortices, with a resolution equivalent to around 0.01° latitude (around 10 km) per pixel.

### 2.1 Velocity Measurements

Horizontal velocities were obtained using semi‐automated image correlation methods (i.e., involving some manual intervention, see Hueso et al., 2009; Sánchez‐Lavega et al., 2019, for details) between pairs of images separated in time by intervals of approximately 1–10 hr. The correlation algorithm used pixel box sizes of 23 × 23 (in the north) or 25 × 25 (in the south), leading to a spatial resolution of the velocity vectors equivalent to around 1° latitude or 1,000 km outside the polar vortices, reducing to around 0.2° or 200 km within the polar vortices themselves. The automatically generated velocity vectors were supplemented by a small number (around 1% of the total) of vectors obtained manually from the motion of visually identified cloud tracers. The estimated measurement uncertainty on each vector was around 5–10 m s^−1^.

**Figure 2:**
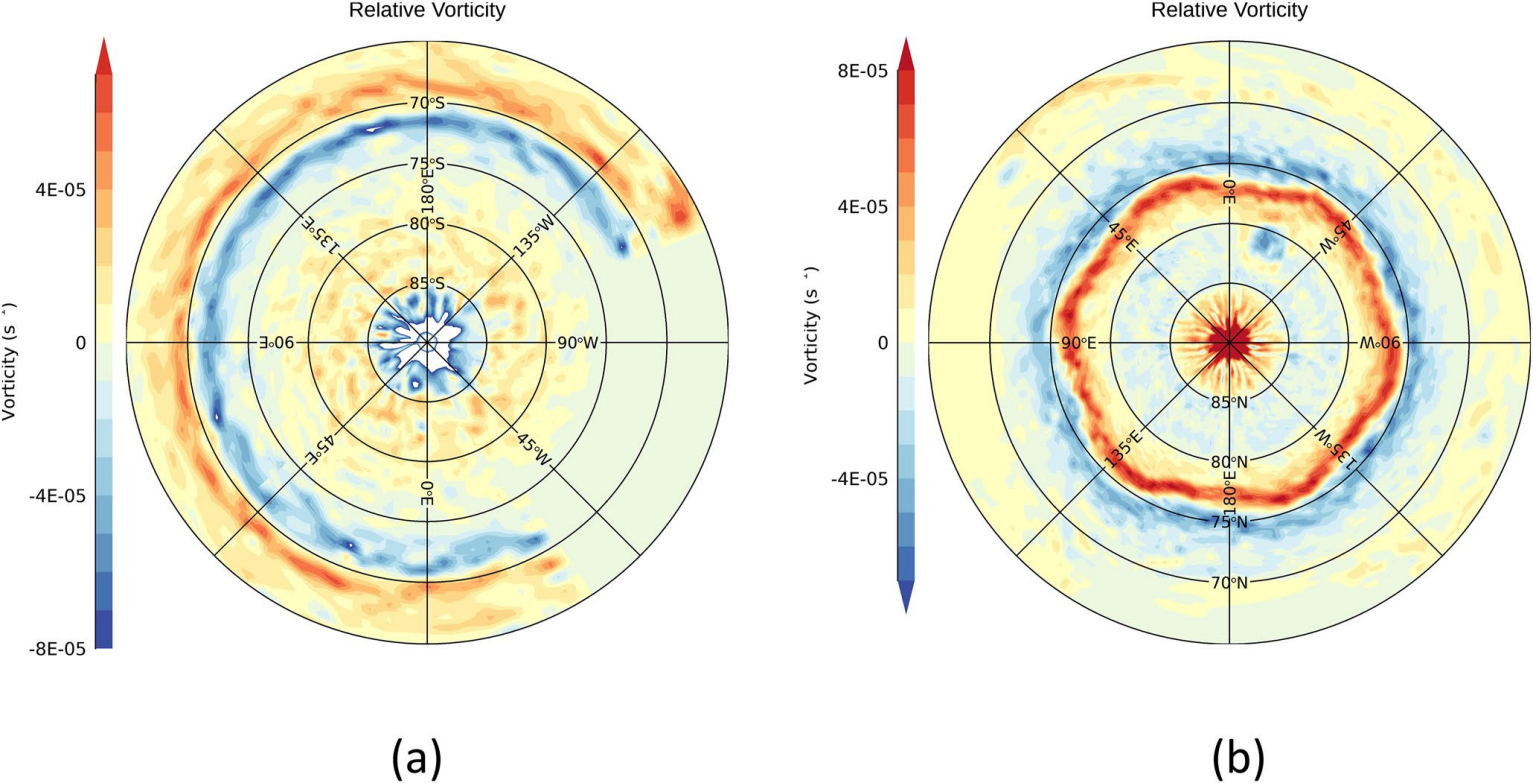
Cloud‐top level vorticity fields, obtained from cloud‐tracked wind measurements using Cassini Imaging Sub‐System images by Antuñano et al. (2015), for Saturn's (a) south polar and (b) north polar regions. Note that the right hand rule is assumed, so red colors imply cyclonic vorticity in the northern hemisphere and anticyclonic in the south, and vice versa. Note also that velocity vectors were not available in the south equatorwards of 76°S between longitudes of ∼35°–110°W.

Figure 2 shows the maps of the relative vorticity in (a) the southern and (b) the nortthern hemispheres. These maps clearly show the regular, symmetrical NPH feature centered on the eastward jet at 76°N, the corresponding near‐circular eastward jet centered at 71°S and the intense cyclonic polar vortices in each hemisphere. Zonal motion at intermediate latitudes is generally westward (relative to Saturn's System III; Desch and Kaiser (1981); Seidelmann et al. (2007); Archinal et al. (2018)) but less strongly concentrated into clear jets. For the present study, the original velocity vectors from Antuñano et al. (2015) were interpolated onto a regular latitude‐longitude grid using convex hulls and Delauney triangulation via the QHULL routine (Barber et al., 1996) of the Interactive Data Language (IDL). The final data set was held on a grid separated by 3° (N) or 4° (S) in longitude and 0.23° (N) or 0.33° (S) in latitude. This almost certainly leads to some oversampling in latitude outside the polar vortices, so fields were typically smoothed to a latitudinal resolution of around 1° for some calculations. See the Supporting Information S1 for further information on the distribution of measured and interpolated velocity vectors and the raw velocity fields.

### 2.2 Errors and Uncertainties

Sources of error and uncertainty in the velocity measurements were discussed by Antuñano et al. (2015). Principal sources of error considered were due to a combination of navigation uncertainties (uncertainties in locating and orienting each image used for correlation) and individual pixel errors. A number of different images were used to obtain these velocity measurements, including both wide and narrow angle cameras on the Cassini orbiter at various viewing and phase angles (see Antuñano et al., 2015, their Table 2), so it is not straightforward to take into account differences in uncertainty in different locations. Navigation errors were estimated to be between 2 and 4 m s^−1^ in most cases, while pixel errors were estimated to be between 1 and 10 m s^−1^ from the effective horizontal resolution and time differences between image pairs. Navigation and pixel errors are expected to be uncorrelated and so here we combine these errors in quadrature and follow Antuñano et al. (2015) in estimating the effective uncertainty in individual velocity vectors to lie within the range 5–10 m s^−1^. This does not, however, take account of the effects of interpolating onto a regular latitude‐longitude grid.

**Figure 3:**
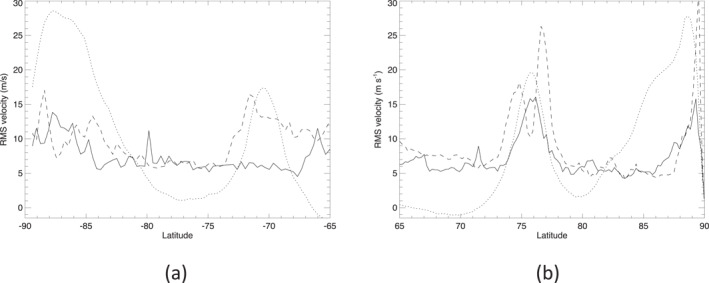
Profiles of root mean square values of *u*′ (dashed lines) and *v*′ (solid lines) for Saturn's (a) south and (b) north polar regions. Scaled profiles of the zonal mean wind $\bar{u}$ are shown dotted for reference.

Figure 3 shows profiles of the individual root mean square velocity components of *u*′ and *v*′, designated as *δu*′ and *δv*′, following subtraction of the zonal mean components $\left(\bar{u},\bar{v}\right)$. This clearly shows increases in both *δu*′ and *δv*′ in the vicinity of the north and south polar jets and the polar vortices, with *δu*′ typically somewhat greater than *δv*′ in these regions. Elsewhere, *δu*′ and *δv*′ take on background values where *δu*′ ≃ *δv*′ ≃ 5–6 m s^−1^. We interpret this to suggest that the isotropic background fluctuations in *u*′ and *v*′ well away from major jets or polar vortices are dominated by measurement noise, suggesting nominal values of measurement error *σ*_*u*′_ ≃ *σ*_*v*′_ ≤ 6 m s^−1^. For the purposes of propagating velocity uncertainties into other derived quantities, therefore, hereafter we take 6 m s^−1^ to be the typical estimate of error in each velocity component.

### 2.3 Zonal Mean Velocities

**Figure 4:**
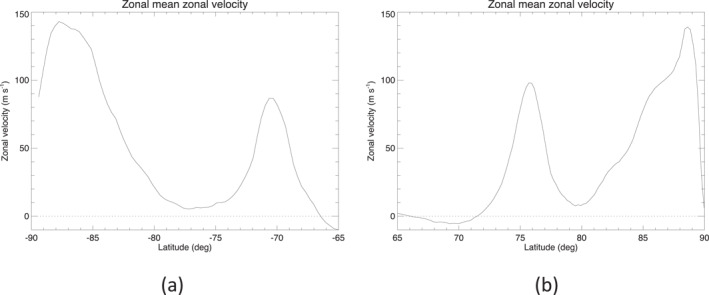
Zonal mean zonal velocity profiles, obtained from Cassini Imaging Sub‐System images by Antuñano et al. (2015) and reinterpolated in the present work onto a regular longitude‐latitude grid, for Saturn's (a) south polar and (b) north polar regions.

The use of a regular latitude‐longitude grid makes it easier, among other things, to compute zonal averages. Figure 4 shows profiles of the zonal mean zonal velocity $\bar{u}$ in (a) the south and (b) the north, computed from the velocities on the new longitude‐latitude grid. This clearly shows the strong eastward jets at 76°N and 71°S and the complex profile across the polar vortices. Both sets of jets are well resolved, with peak velocities of the North and South Polar Jets (NPJ and SPJ) around 100 and 80 m s^−1^ respectively. The zonal mean structure of the polar vortices indicates peak velocities of around 140 m s^−1^ in both hemispheres with complex “shoulders” on the equatorward side of each vortex that differ markedly between the north and south. This is slightly weaker in the south than shown by Antuñano et al. (2015) and Dyudina et al. (2009), likely due to some implicit smoothing in the interpolation used here to a somewhat lower resolution compared to the earlier studies.

### 2.4 Eddy Kinetic Energy

**Figure 5:**
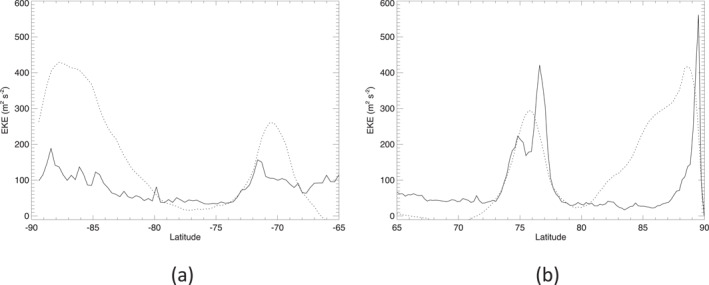
Profiles of eddy kinetic energy $\left(K_{E}=1/2\left(\bar{u^{\prime 2}+v^{\prime 2}}\right)\right)$ for Saturn's (a) south and (b) north (b) polar regions. Scaled profiles of the zonal mean wind $\bar{u}$ are shown dotted for reference.

On subtracting the zonal mean velocities from the original velocity field, we can then calculate variances and covariances of the residual eddy components. Figure 5 shows the profiles of specific eddy kinetic energy (EKE) (neglecting any horizontal density variations), defined as

(1) $$K_{E}=\frac{1}{2}\left(\bar{u^{\prime 2}}+\bar{v^{\prime 2}}\right),$$

as a function of latitude in each hemisphere, where primed quantities represent departures from the zonal mean (denoted by the overbar). This exhibits markedly different behavior between each hemisphere, with much larger peak values of *K*_*E*_ in the north compared with the south. In particular, there is a pronounced double peak in *K*_*E*_ centered on the latitude of the NPJ, corresponding to the strong NPH hexagonal wave that modulates both *u* and *v* in longitude. An even stronger peak in *K*_*E*_ exceeding 500 m^2^ s^−2^ is seen at the inner edge of the North Polar Vortex (hereafter NPV), indicating a strong departure of the vortex from a circular shape. Although a somewhat similar trend is seen with the south polar vortex it is much weaker (<200 m^2^ s^−2^) and more widely spread in latitude. These apparent peaks so close to each pole might be accentuated by possible small systematic errors in location due to the interpolation method used here, although this is hard to quantify. There is also evidence for a weak and broad peak in *K*_*E*_ around the latitude of the SPJ but mostly <100 m^2^ s^−2^. Despite these differences, the area‐weighted average values of *K*_*E*_ in both hemispheres are remarkably similar (76.5 ± 0.8 J kg^−1^ in the north and 80.0 ± 0.8 J kg^−1^ in the south) and represent around 10% of the total horizontal KE in either hemisphere.

### 2.5 Eddy Length Scales

Given profiles of *K*_*E*_ we can then calculate estimates of quantities such as the Rhines wavelength scale *λ*_*R*_, representing a cross‐over scale between large‐scale waves and small‐scale turbulence (e.g., Chemke & Kaspi, 2015; Vallis, 2017; Vasavada & Showman, 2005) and defined in terms of *K*_*E*_ by

(2) $$\lambda_{R}≃2\pi {\left(\frac{\sqrt{K_{E}}}{\beta }\right)}^{1/2},$$

where *β* = (1/*a*)*df*/*dϕ* is the northward gradient of the Coriolis parameter, *f* = 2Ω sin *ϕ*, with latitude *ϕ*. This typically represents a scale comparable to the distance between eastward or westward zonal jet maxima in geostrophic turbulence (e.g., Chemke & Kaspi, 2015; Vallis, 2017; Vasavada & Showman, 2005). This scale may also be compared with other length scales, such as Saturn's mean radius (*a* = 5.823 × 10^4^ km) and scales representative of energetic eddies, such as the first baroclinic Rossby radius of deformation, *L*_*D*_. The latter is defined as a wavelength here by

(3) $$\lambda_{D}=2\pi L_{D}≃2\pi \left(\frac{NH}{f}\right),$$

where *N* is the mean buoyancy or Brunt‐Väisälä frequency, *H* is a vertical scale height (often taken somewhat arbitrarily to be the pressure scale height near 1 bar pressure). For Saturn, *N* is not well measured beneath the visible clouds though likely varies greatly with depth, and *H* is also not known with much confidence. *L*_*D*_ was estimated by Read et al. (2009) from measurements of Saturn's potential vorticity configuration near the cloud tops to vary approximately with latitude as *L*_*D*_ ≃ 1,500/sin *ϕ* km, so here we take

(4) $$\lambda_{D}≃3000\pi /|\sin\phi |\;km.$$

**Figure 6:**
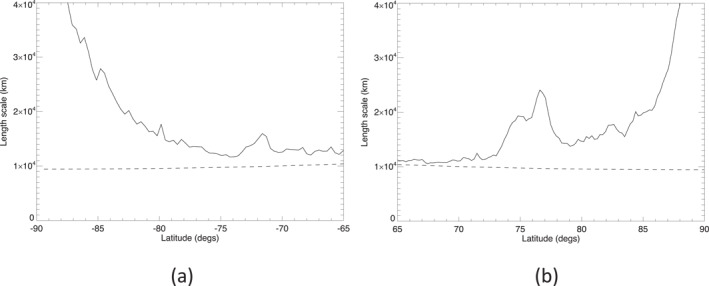
Key lengthscales computed for Saturn's south polar (a) and north polar regions (b). Solid line is the Rhines wavelength scale *λ*_*R*_, while the dashed line shows estimates of the wavelength *λ*_*D*_ corresponding to the first baroclinic Rossby radius of deformation *L*_*D*_ (see text).

Profiles of *λ*_*R*_ and *λ*_*D*_, calculated using Equations 2 and 4, are shown in Figure 6 for (a) the north and (b) the south. These show that both *λ*_*R*_ and *λ*_*D*_ are mostly much smaller than the planetary radius *a* and indicate how *λ*_*R*_ diverges to very large scales as each pole is approached (since *β* → 0 as |*ϕ*| → 90°), while *λ*_*D*_ increases slowly with *ϕ* away from the pole. *λ*_*R*_ and *λ*_*D*_ are comparable around latitude *ϕ* ∼ 60–65° in each hemisphere, indicating that *λ*_*D*_ may tend to be similar to or even larger than *λ*_*R*_ equatorward of around 60° (cf. Chemke & Kaspi, 2015, their Figure 4). There are local variations in *λ*_*R*_, however, especially close to the NPJ, indicating that variations in *λ*_*D*_/*λ*_*R*_ may be found elsewhere. But in general this suggests that Saturn's mid‐high latitude regions are characterized by values of *λ*_*D*_ that are smaller than *λ*_*R*_. It is also of interest to note that *λ*_*R*_ is comparable to the separation distance between the NPJ and SPJ and the adjacent eastward jets on the equatorward sides. *λ*_*D*_ at 76°N is around 10^4^ km and corresponds to a longitudinal wavenumber of around *m* = 9 and is somewhat larger than the FWHM of the NPH at around 5,800 km.

## 3 Analysis Methods

In this section we outline the diagnostics used to examine the properties of the polar circulations on Saturn, with particular reference to the transfer of KE between different scales of motion.

### 3.1 Eddy‐Zonal Flow Interactions

The forcing of zonal jets by eddies is commonly discussed in terms of the zonal mean zonal momentum equation, which can be written

(5) $$\frac{\partial \bar{u}}{\partial t}-\left(f+\bar{\zeta }\right)\bar{v}+\bar{w}\frac{\partial \bar{u}}{\partial z}=-\frac{1}{\rho_{0}}\nabla .F_{m}+\bar{F},$$

(e.g., Andrews et al., 1987), where $\bar{\zeta }$ is the vertical component of zonal mean vorticity, $\bar{v}$ and $\bar{w}$ are the zonal mean meridional and vertical velocity components (where $\bar{v}>0$ is northward in both hemispheres) and $\bar{F}$ represents frictional effects and body forces acting on the flow. **F**_**m**_ represents the eddy flux of zonal momentum in the meridional (*ϕ*, *z*) plane due to the Reynolds stresses. In spherical coordinates, ∇.**F**_**m**_ can be written

(6) $$\frac{1}{\rho_{0}}\nabla .F_{m}=-\frac{1}{a\;\cos^{2}\;\phi }\frac{\partial }{\partial \phi }\left(\bar{u^{\prime }v^{\prime }}\cos^{2}\phi \right)-\frac{1}{\rho_{o}}\frac{\partial }{\partial z}\left(\rho_{0}\bar{u^{\prime }w^{\prime }}\right).$$

where *ρ*_0_(*z*) is a background reference density profile, so **F**_**m**_ becomes

(7) $$F_{m}=-\rho_{0}\;\cos\;\phi \left[\bar{u^{\prime }v^{\prime }},\bar{u^{\prime }w^{\prime }}\right].$$

For the present problem we have no direct information on vertical velocity, other than to anticipate that it is likely to be much smaller than typical horizontal velocities (by a factor O(*Ro*.*H*/*L*), where *Ro* is the Rossby number and *H* and *L* are vertical and horizontal lengthscales). So we will focus here on the horizontal eddy fluxes and the Reynolds stress divergence contribution to the energy budget.

The rate of conversion of KE between eddies and zonal mean flow is typically calculated from Equation 5, integrating in latitude (and height) across the domain. Neglecting the vertical dimension in the present context, we can calculate the rate of increase of zonal mean KE, denoted by *K*_*Z*_ as.

(8) $$\frac{dK_{Z}}{dt}=C\left(K_{E},K_{Z}\right)$$

(9) $$=-\frac{\int \left[\frac{\bar{u}}{a\;\cos\;\phi }\right]\frac{\partial }{\partial \phi }\left(\bar{u^{\prime }v^{\prime }}\cos^{2}\phi \right)d\phi }{\int \cos\;\phi d\phi }$$

(10) $$=\frac{\int \frac{\partial }{\partial \phi }\left[\frac{\bar{u}}{a\cos\phi }\right]\bar{u^{\prime }v^{\prime }}{\cos\;}^{2}\phi d\phi }{\int \cos\;\phi d\phi },$$

neglecting boundary terms in the usual way (cf. Peixóto & Oort, 1974), where *C*(*K*_*E*_, *K*_*Z*_) represents the corresponding conversion rate of eddy KE (*K*_*E*_) to *K*_*Z*_.

### 3.2 Errors and Uncertainties in $\bar{u^{\prime }v^{\prime }}$ and *C*(*K*_*E*_, *K*_*Z*_)

Uncertainties in the values of *C*(*K*_*E*_, *K*_*Z*_) determined via Equation 9 or Equation 10 are likely to be dominated by uncertainties in $\bar{u^{\prime }v^{\prime }}$ associated with velocity errors *σ*_*u*′_ and *σ*_*v*′_, which are relatively larger than those in $\bar{u}$ (cf. Ingersoll et al., 1981). In estimating uncertainties in $\bar{u^{\prime }v^{\prime }}$ we follow Ingersoll et al. (1981), their Eq (7), assuming errors in *u*′ and *v*′ to be uncorrelated. Thus

(11) $$\sigma^{2}\left(\bar{u^{\prime }v^{\prime }}\right)≃\left(\sigma_{u^{\prime }}^{2}\delta u^{\prime 2}+\sigma_{v^{\prime }}^{2}\delta u^{\prime 2}+\sigma_{u^{\prime }}^{2}\sigma_{v^{\prime }}^{2}\right)/n,$$

where *n* is the number of velocity points in longitude used to calculate the momentum flux. This is an approximation since we assume *δu*′ and *δv*′ to represent the true signal even though they are actually contaminated by measurement noise. But this does at least provide an upper limit on the error in $\bar{u^{\prime }v^{\prime }}$ as $\sigma \left(\bar{u^{\prime }v^{\prime }}\right)$.

For estimating uncertainty in the integrand of *C*(*K*_*E*_, *K*_*Z*_) using Equation 10 (hereafter designated *c*(*K*_*E*_, *K*_*Z*_)), we follow Ingersoll et al. (1981) in neglecting the uncertainty in $d\bar{u}/dy=\cos\;\phi \;d/d\phi \left[\bar{u}/\left(a\cos\phi \right)\right]$ to obtain

(12) $$\sigma \left(c\left(K_{E},K_{Z}\right)\right)=\frac{\partial }{\partial \phi }\left[\frac{\bar{u}}{a\;\cos\;\phi }\right]\sigma \left(\bar{u^{\prime }v^{\prime }}\right)\cos\;\phi ,$$

for a particular latitude *ϕ*. The standard error in *C*(*K*_*E*_, *K*_*Z*_), averaged over a range in latitude, is then given by

(13) $$\sigma \left(C\left(K_{E},K_{Z}\right)\right)=\frac{\int \frac{\partial }{\partial \phi }\left[\frac{\bar{u}}{a\;\cos\;\phi }\right]\sigma \left(\bar{u^{\prime }v^{\prime }}\right)\cos^{2}\;\phi d\phi }{\sqrt{p}\int \cos\;\phi d\phi },$$

where *p* is the number of latitude rows across the region of interest.

### 3.3 Spectral Decomposition

The formulation above considers just the interaction between the zonal jet flow and non‐axisymmetric eddies of all scales. The *C*(*K*_*E*_, *K*_*Z*_) term can, however, be decomposed further into contributions from different zonal harmonics of wavenumber index *m* via a Fourier analysis of *u*′ and *v*′ in longitude (cf. Chemke & Kaspi, 2015). Given the complex amplitude spectra of *u*′ and *v*′, denoted here by $\tilde{u^{\prime }}$ and $\tilde{v^{\prime }}$, the relevant self‐interaction component of the Reynolds stress becomes

(14) $$\tilde{u^{\prime }v^{\prime }}\left(m,\phi \right)=\tilde{u^{\prime }}\left(m,\phi \right){\tilde{v^{\prime }}}^{*}\left(m,\phi \right)+{\tilde{u^{\prime }}}^{*}\left(m,\phi \right)\tilde{v^{\prime }}\left(m,\phi \right),$$

where starred quantities represent complex conjugates. We can thus obtain the spectrally decomposed eddy‐zonal KE conversion rate by extension of Equation 9 using Equation 14,

(15) $$\tilde{C\left(K_{E},K_{Z}\right)}\left(m\right)=\frac{\int \left[\frac{\bar{u}}{a\;\cos\;\phi }\right]\partial /\partial \phi \left(\tilde{u^{\prime }v^{\prime }}\left(m,\phi \right)\cos^{2}\;\phi \right)d\phi }{\int \cos\;\phi d\phi },$$

In our analyses below, therefore, we include computations of both *C*(*K*_*E*_, *K*_*Z*_) and the integrand $\tilde{c\left(K_{E},K_{Z}\right)}\left(m\right)$, integrated over various ranges in latitude and locally as a function of *ϕ*. Note that, for the southern hemisphere, the gap in longitude coverage of the wind measurements between 35° and 110° was filled by copying a segment of data from another interval in longitude. This was necessary to enable the use of Fast Fourier methods to compute zonal spectra. The sensitivity of quantities such as $\tilde{c\left(K_{E},K_{Z}\right)}\left(m\right)$ to the range of longitudes used to fill the gap was evaluated by trying different longitude segments and found to be small compared with the estimated measurement uncertainties.

Uncertainties in $\tilde{C\left(K_{E},K_{Z}\right)}\left(m\right)$ were estimated in a similar way to Equation 13, but in which the errors were spread with respect to wavenumber *m* assuming errors in each wavenumber were uncorrelated with every other. This is ensured by defining

(16) $$\sigma^{2}\left(C\left(K_{E},K_{Z}\right)\right)=\sum_{m}\sigma^{2}\left(\tilde{C\left(K_{E},K_{Z}\right)}\right)\left(m\right).$$

In the absence of more detailed information, we assume for simplicity that uncertainties are similar in magnitude at all scales, so the error estimate *σ*(*C*(*K*_*E*_, *K*_*Z*_)) is distributed evenly across all wavenumbers, even though it is likely, for example, that navigation errors are correlated on large scales whereas pixel errors are uncorrelated. It was not possible to track these errors in detail between different image pairs but readers should be aware that errors may actually be larger at small *m* than for higher wavenumbers.

## 4 Eddy‐Zonal Flow Interactions

In this section we present the results of analyzing the rates of conversion between eddy and zonal mean KE in the vicinity of both polar regions of Saturn. Calculations include both the total conversion rate averaged over the whole polar region |*ϕ*| > 65° and particular subranges of *ϕ* to focus on both polar vortices and the NPJ and SPJ.

### 4.1 Total Conversion Rates

**Figure 7:**
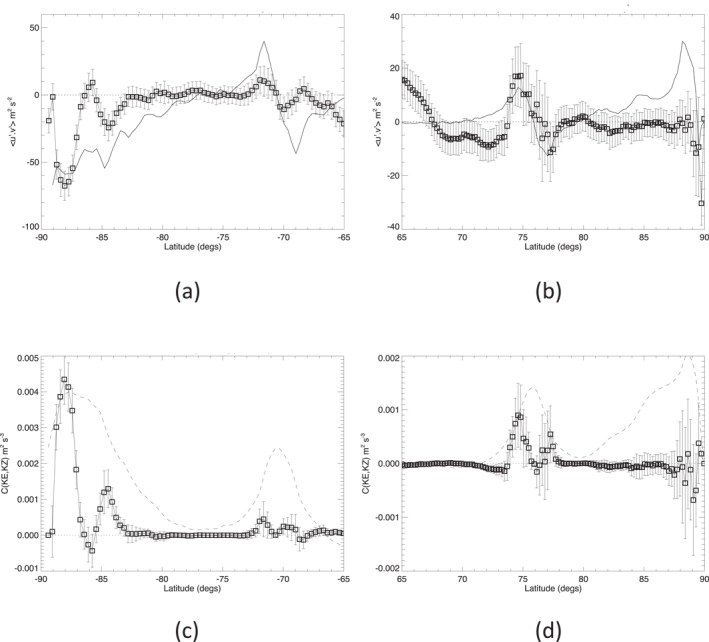
(a and b) Profiles of eddy momentum flux, $\bar{u^{\prime }v^{\prime }}$, smoothed in latitude to a resolution of 1° for Saturn's (a) south polar and (b) north polar regions. Solid lines in (a and b) show the corresponding profiles of $d/d\phi \left(\bar{u}/\cos\phi \right)$ while scaled profiles of $\bar{u}$ are shown dashed in (c) and (d). Note that different scales are used for the axes in the plots in (a and b) to show the features more clearly. (c and d) Profiles of KE conversion rate *c*(*K*_*E*_, *K*_*Z*_) as given by the integrand of Equation 10 for the southern (c) and northern (d) polar regions.

Given the gridded velocity fields described in Section 2 above, it is straightforward to compute the northward flux of eddy momentum, $\bar{u^{\prime }v^{\prime }}$, at each latitude row to obtain the profiles presented in Figures 7a and 7b. The unfiltered/unsmoothed results are somewhat noisy, as is clear from the error estimates shown by the error bars in Figures 7a and 7b, computed from Equation 11, and the correlation coefficients between *u*′ and *v*′; see Figure S4 in the Supporting Information S1. But there are clear features, coherent in latitude, in the profiles at the locations of the south polar vortex and around the latitudes of the north and south polar jets. Figures 7a and 7b also show solid line profiles of the zonal mean wind gradient $d\bar{u}/dy$ in each hemisphere for reference. This shows some complex structure around the polar vortices, but with clear changes of sign of $\bar{u^{\prime }v^{\prime }}$ close to the cores of both the NPJ and SPJ.

Calculating nominal values of the integrand *c*(*K*_*E*_, *K*_*Z*_) from Equation 10, without any explicit smoothing in latitude, we obtain the mean local KE conversion rate from eddies into the zonal jet, with the results shown in Figures 7c and 7d. Error bars represent the estimated uncertainty according to Equation 12 and indicate clear regions of strong eddy‐zonal flow interactions in the south polar vortex and on either side of the jet cores at 69°–73°S and 74°–78°N. The results indicate a significant positive conversion from eddies to zonal flow within the NPJ and SPJ, and also within the South Polar Vortex (SPV) polewards of 83°S. The pattern of *c*(*K*_*E*_, *K*_*Z*_) in the North Polar Vortex (NPV), however, looks more complicated and noisy, with no obvious direction of energy conversion.

Integrating these local conversion rates over the whole polar domain in each hemisphere using Equation 9, we obtain the overall mean conversion rates shown in the first two rows of Table 1. This shows a general trend for eddies to be transferring KE into the zonal jets in both polar regions, though at around three times the rate in the south compared with the north, at least at the time when these observations were acquired. The uncertainties are estimated as discussed in Section 3.2, Equation 13.

**Table 1:** Eddy‐Zonal Flow Kinetic Energy Conversion Rates on Saturn, Computed Over Different Latitude Ranges Using the Area‐Weighted Mean of the Lorenz Form Defined in Equation 10 and the Local Reynolds Stress Divergence Defined in Equation 9 From the Data Set of Antuñano et al. (2015)

Feature	Latitude range (°)	*C*(*K*_*E*_, *K*_*Z*_) (W kg^−1^)
North polar region	66°–90°N	4.3 ± 2.3 × 10^−5^
South polar region	66°–90°S	1.4 ± 0.3 × 10^−4^
North polar jet	70°–79°N	1.0 ± 0.5 × 10^−4^
South polar jet	66°–76°S	8.7 ± 3.7 × 10^−5^
North polar vortex	80°–90°N	−4.2 ± 3.6 × 10^−5^
South polar vortex	80°–90°S	4.7 ± 0.6 × 10^−4^

### 4.2 Regional Conversion Rates

If we focus attention on particular features or regions, it is of interest to evaluate the contribution of the northern and southern polar jets and the polar vortices to the overall transfer of KE from eddies to zonal flow in each polar region. The juxtaposition of the peaks and troughs of $\bar{u^{\prime }v^{\prime }}$ in Figures 7a and 7b with the profile of $d\bar{u}/dy$ suggest a possible local correlation between $\bar{u^{\prime }v^{\prime }}$ and $d/d\phi \left(\bar{u}/\cos\phi \right)$, especially in the vicinity of the NPJ and SPJ, consistent with a positive contribution to *C*(*K*_*E*_, *K*_*Z*_) (cf. Equation 10).

Also shown in Table 1 are the values of *C*(*K*_*E*_, *K*_*Z*_) computed over latitude ranges centered respectively on the zonal mean polar jets and vortices. For the polar jets, centered respectively at around 76°N and 70°S, *C*(*K*_*E*_, *K*_*Z*_) is strongly positive, indicating a relatively powerful local transfer of KE from eddies into each jet at a level of order 10^−4^ W kg^−1^. For these features, the conversion rate into the NPJ is somewhat larger than in the SPJ and somewhat larger in the north than the average across the rest of the north polar region. This is in contrast to the south where the conversion rate into the SPJ is similar to or slightly less than the average across the south polar region. From this calculation, however, it is not clear which scale of nonzonal eddies or waves might be determining the overall rate of KE transfer into the zonal mean zonal jets. In particular, the role of the wavenumber *m* = 6 meanders in the NPH in these transfers is not clear since there are evidently waves of many differing zonal wavenumbers present across both regions.

For the polar vortices, the calculations of *C*(*K*_*E*_, *K*_*Z*_) reveal major differences between the North Polar Vortex (NPV) and the South Polar Vortex (SPV), at least so far as their energetics are concerned. For the NPV, *C*(*K*_*E*_, *K*_*Z*_) is seen in Table 1 to be small and negative with a value around −4.2 ± 3.6 × 10^−5^ W kg^−1^. This would suggest that eddies are gaining just a little KE at the expense of the zonally symmetric zonal flow in the vortex, perhaps marginally suggestive of a barotropic instability though with relatively large uncertainty. Such an instability would not be unduly surprising, for example, if such polar vortices were dynamically similar in some respects to the cores of tropical cyclones on Earth, leading to the growth of elliptical or even polygonal distortions of the main vortex. For the South Polar Vortex (SPV), however, *C*(*K*_*E*_, *K*_*Z*_) is seen in Table 1 to be strongly positive when integrated over the entire SPV poleward of 80°S with relatively high statistical significance. However, Figure 7a indicates that the conversion rate varies a lot with latitude with strong convergence of eddy momentum fluxes near latitudes of 85° and 89°S and divergent fluxes (indicative of local westward forcing of zonal flow) around 87° and 84°S. This would seem to suggest that parts of the axisymmetric southern polar vortex were gaining energy from non‐axisymmetric eddies while other parts of the vortex were losing energy, although more information, for example, on the structure of flow, may be desirable to interpret this result.

### 4.3 Spectral Decomposition

Although the simple partitioning of the flow between zonally symmetric and non‐axisymmetric components allows us to determine the overall rate of KE conversion between eddies and zonal jets, this approach integrates over all eddy length scales. As a result it does not provide much insight into the roles of eddies of different lengthscales in either driving or feeding barotropically off of the zonal jets. As outlined in Section 3.1 above, however, we can further decompose the flow into its zonal harmonics and thereby examine the contribution of each zonal wavenumber to the overall energy budget for the zonal jets.

**Figure 8:**
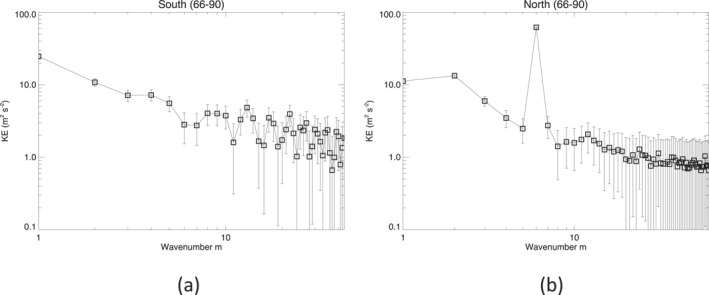
Area‐weighted kinetic energy spectra for (a) the southern and (b) the northern polar regions (66°–90° latitude). An alternative version of this figure with linear scales on the axes is presented as Figure S5 in the Supporting Information S1.

Although the NPH feature is prominent in the northern polar regions, the area‐averaged zonal KE spectrum (see Figure 8b) shows that KE is present at all zonal wavenumbers that are resolved in the observations. Thus, we see in the north a sloping continuum in the spectrum of KE with increasing *m*, leading into a fairly clear noise floor (cf. the estimated error bars) for *m* ≳ 20, upon which is superposed a strong peak at *m* = 6 representing the NPH. In the south, however, the spectrum appears flatter and somewhat weaker overall than in the north at low wavenumbers (see Figure 8a) but still with significant eddy kinetic energy (EKE) stretching to some higher wavenumbers above the noise floor of around 1–2 J kg^−1^ per wavenumber.

**Figure 9:**
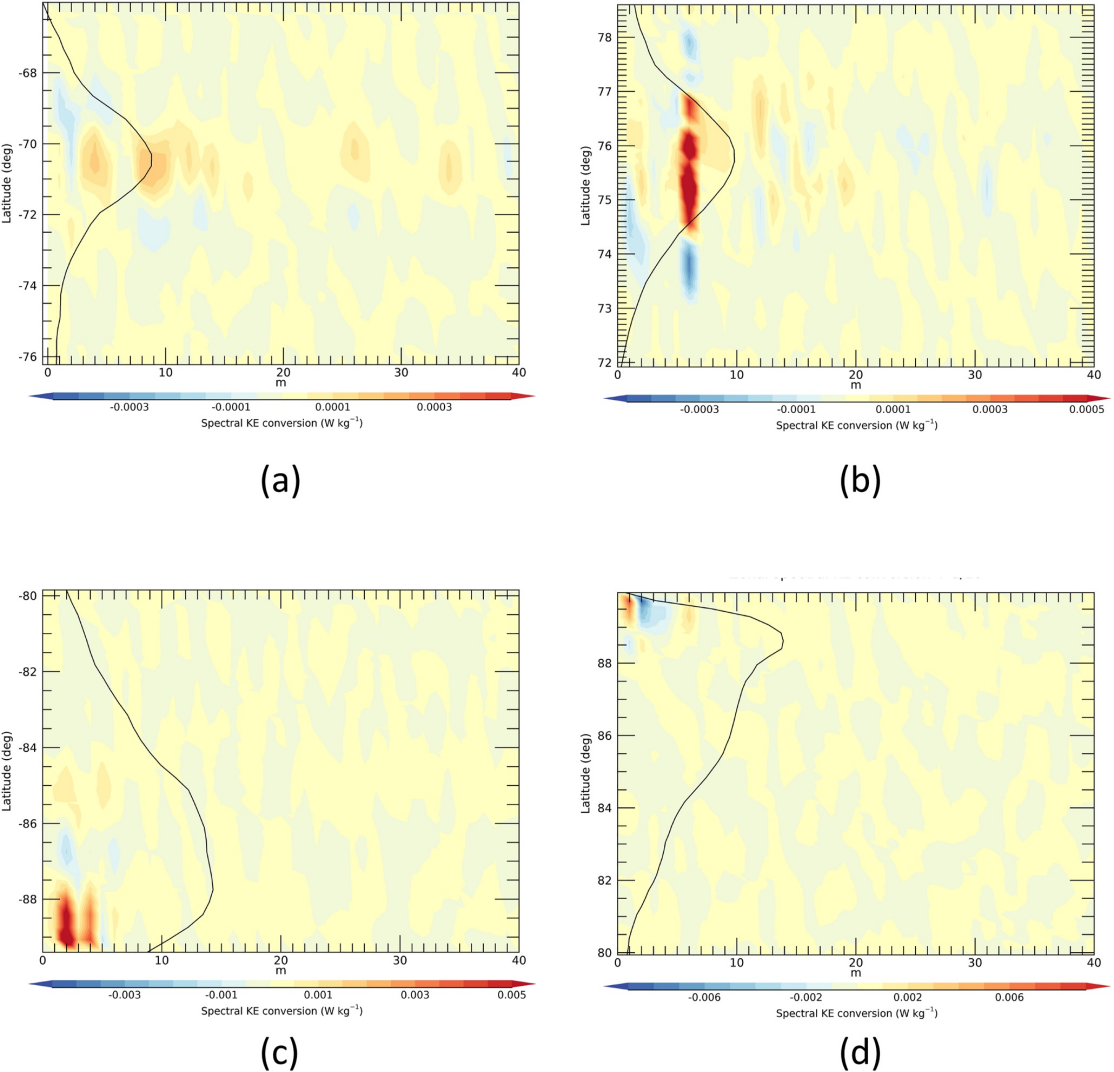
Spectrally resolved, local eddy‐zonal flow KE conversion rate, given by Equation 17, $\tilde{c\left(K_{E},K_{Z}\right)}\left(m,\phi \right)$, versus zonal wavenumber *m* and latitude *ϕ*, for (a) Saturn's south polar jet, (b) north polar jet, (c) south polar vortex and (d) north polar vortex. Note the difference in color scales between each frame.

Decomposing *C*(*K*_*E*_, *K*_*Z*_) into its zonal harmonics using Equation 15 we can quantify the contributions to the zonal mean KE budget due to different zonal wavenumber components. Figure 9 shows the integrand of the numerator of Equation 15,

(17) $$\tilde{c\left(K_{E},K_{Z}\right)}\left(m,\phi \right)=\left[\frac{\bar{u}}{a\;\cos^{2}\;\phi }\right]\frac{\partial }{\partial \phi }\left(\tilde{u^{\prime }v^{\prime }}\left(m,\phi \right)\cos^{2}\;\phi \right),$$

as a function of both zonal wavenumber *m* and latitude *ϕ* for each of the north and south polar jets and polar vortices. $\tilde{c\left(K_{E},K_{Z}\right)}\left(m,\phi \right)$ for the SPJ for shows a broadly positive local conversion of eddy to zonal KE over a wide range of zonal harmonics, centered on the jet core, with weaker negative conversions on the flanks of the zonal jet. In contrast, the equivalent local conversion of eddy KE into the NPJ is clearly dominated by the contribution from the *m* = 6 hexagonal wave (Figure 9a), with a strong positive contribution into the jet core and weaker negative contributions on both its northern and southern flanks. This indicates clearly that the *m* = 6 component of the hexagon wave itself is feeding KE into the zonal mean NPJ, tending to accelerate its core and decelerating the flanks, thereby tending to sharpen the eastward jet. Contributions from other zonal harmonics are much weaker and more complicated in latitudinal structure, though a small signal at the first harmonic of the hexagon, *m* = 12, is evident among others with a weak dipolar structure in latitude.

**Figure 10:**
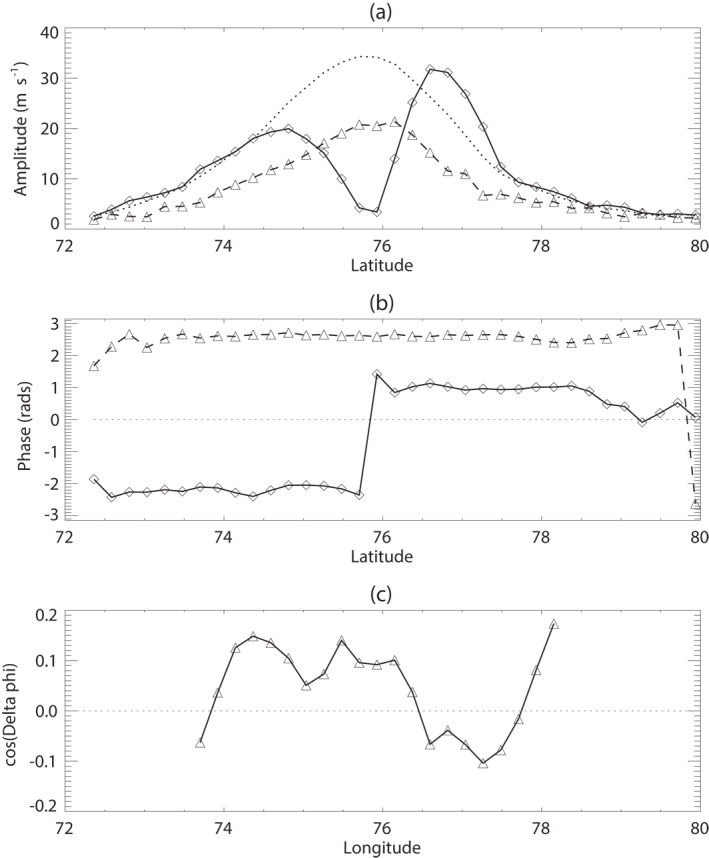
Latitudinal structure of the *m* = 6 component of the north polar hexagon from Fourier decomposition of the north polar wind fields. (a) Amplitude profiles of *u*′ (solid line with diamond points) and *v*′ (dashed line with triangle points) together with scaled profile of the *m* = 0 (zonal mean $\bar{u}$; dotted line); (b) profiles of zonal phase of *m* = 6 for *u*′ (solid line with diamond points) and *v*′ (dashed line with triangle points); (c) profile of cos(*γ*(6, *ϕ*)), representing the cosine of the phase difference between the *m* = 6 components of *u*′ and *v*′ (cf. Equation 20).

The structure of the *m* = 6 component that leads to the upscale conversion of KE into the *m* = 0 zonal jet is shown in Figure 10, which presents the amplitude and phase profiles of *u*′ and *v*′ (Figures 10a and 10b) and their net contribution to $\bar{u^{\prime }v^{\prime }}$ in Figure 10c. This clearly shows *v*′(*m* = 6) peaking in amplitude around the zonal mean jet core while *u*′(*m* = 6) has a double‐peaked structure on the flanks of the zonal mean jet. The phase of *v*′(*m* = 6) seems remarkably constant across the whole region while *u*′(*m* = 6) jumps by approximately *π* at the jet core, consistent with a change of sign of *u*′ on either side of the jet (for a rendering in physical space of the superposition of the *m* = 0 and *m* = 6 components of the velocity field, which accounts for more than 93% of the total KE between 72° and 80°N, see Figure S7 in the Supporting Information S1). The contribution of *m* = 6 to $\bar{u^{\prime }v^{\prime }}$ is determined by the product of the amplitudes of *u*′ and *v*′ and the phase difference between them. Defining

(18) $$u^{\prime }\left(m\right)=U_{m}\left(\phi \right)\cos\left(m\theta +\gamma \left(m,\phi \right)\right)$$

(19) $$v^{\prime }\left(m\right)=V_{m}\left(\phi \right)\cos\left(m\theta \right),$$

where *γ* is the phase difference between *u*′ and *v*′, the contribution of the component *m* to $\bar{u^{\prime }v^{\prime }}$ is given by

(20) $$\bar{u^{\prime }v^{\prime }}\left(m,\phi \right)=\frac{U_{m}\left(m,\phi \right)V_{m}\left(m,\phi \right)}{2}\;\cos\left(\gamma \left(m,\phi \right)\right).$$

**Figure 11:**
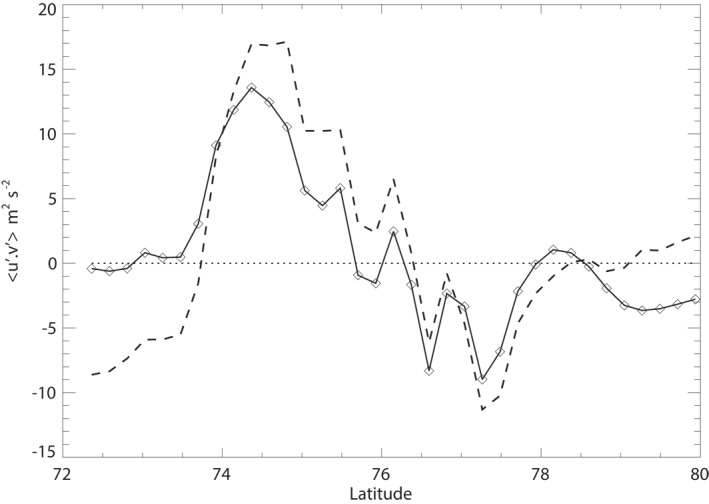
Latitudinal structure of the *m* = 6 contribution to $\bar{u^{\prime }v^{\prime }}$ (solid line with diamonds) in the vicinity of the NPH (cf. Equation 20) from the Cassini velocity measurements. The full profile of $\bar{u^{\prime }v^{\prime }}$ in this region is shown by the dashed line, indicating that *m* = 6 accounts for most of the meridional momentum flux at these latitudes.

The observed structure of the *m* = 6 component of the NPH shows a slight shift in phase difference between *u*′ and *v*′ such that cos(*γ*(6, *ϕ*)) is non‐zero at most latitudes and changes sign across the zonal mean jet core (see Figure 10c). Figure 11 also shows the corresponding profile of $\bar{u^{\prime }v^{\prime }}\left(m=6,\phi \right)$, which has a similar distribution to the total $\bar{u^{\prime }v^{\prime }}$ profile (shown as a dashed line) and evidently accounts for most of the total $\bar{u^{\prime }v^{\prime }}$ due to all resolved zonal harmonics.

**Figure 12:**
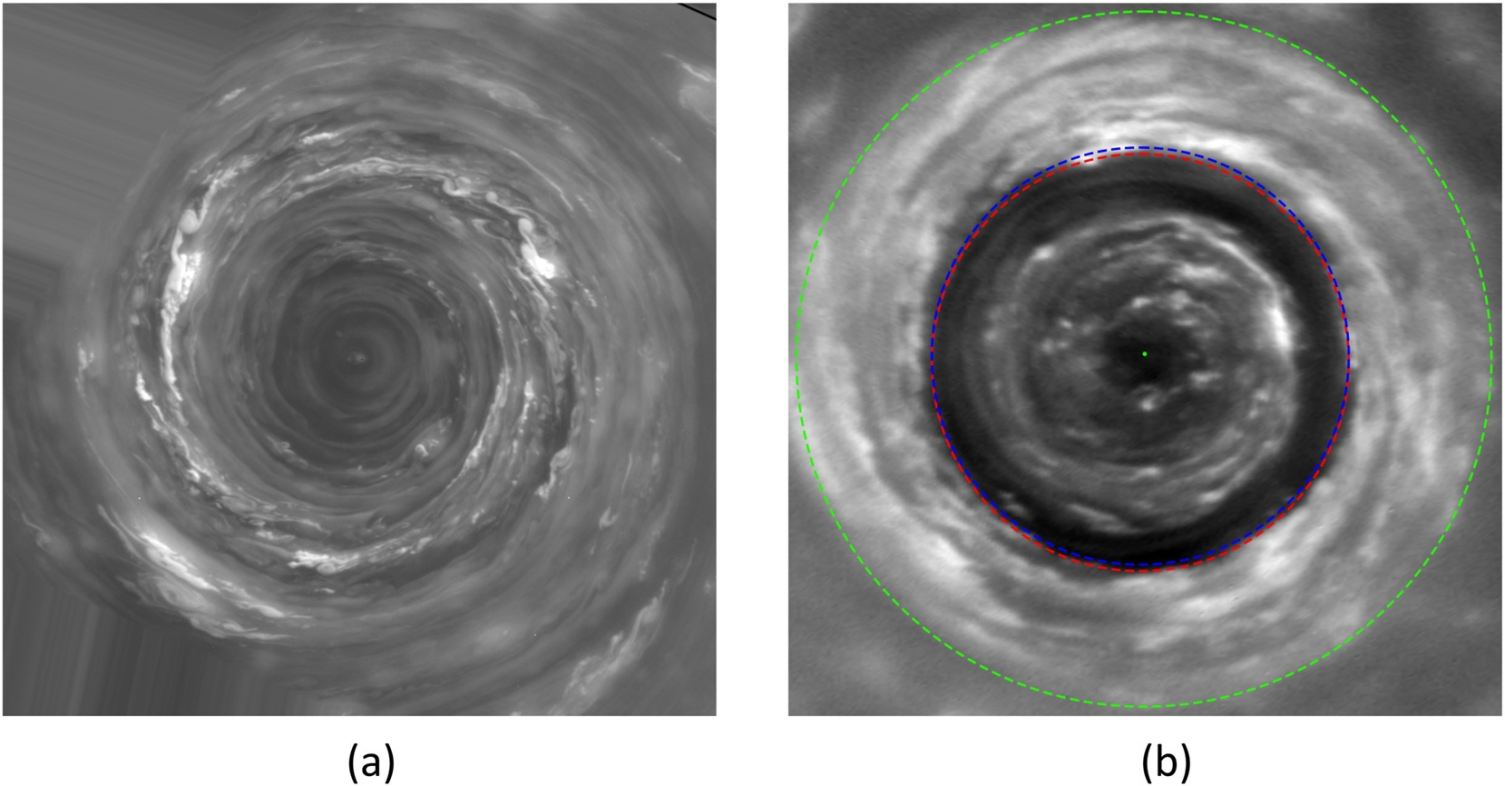
Images of the core of Saturn's North Polar Vortex, obtained by the Cassini Imaging Sub‐System Narrow‐angle camera using the CB2 filter in (a) June 2013 (Image N1749893515_1 (COISS 2083)) and (b) April 2014 (Image N1775155245_1 (COISS 2090)) using the Wide‐angle camera. The image in (b) shows blue and green dashed circles centered on the best estimate of Saturn's north pole (at latitudes of 88.6° and 87.7° N respectively), while the (slightly displaced) red circle is aligned with the approximately circular cloud albedo boundary. Image scale of (a) is 5.3 km per pixel and of (b) is about 17 km per pixel. Image credits from NASA/JPL/Space Science Institute with permission.

$\tilde{c\left(K_{E},K_{Z}\right)}\left(m,\phi \right)$ for the NPV is more complicated (see Figure 9d) but is evidently dominated by contributions from low wavenumbers *m* < 5, particularly very close to the pole. The predominance of a strong contribution from *m* = 1 is somewhat surprising though images of the vortex (e.g., Antuñano et al., 2015; Sayanagi et al., 2017, and Figure 9a) do appear to show some spiral cloud features and occasional secondary vortices that may break its circular symmetry. The significance of *m* = 1, however, might be indicative of a small displacement of the (nearly axiymmetric) vortex away from the assumed position of the pole. Figure 12b shows a Cassini Imaging Sub‐System image of the NPV with blue and green dashed circles centered on the best estimate of the position of Saturn's north pole. The red dashed circle, however, is aligned with the approximately circular cloud albedo boundary and is slightly displaced from the nearby blue latitude circle, which may indicate either a small navigation error or an actual displacement of the NPV from the north pole itself. Other significant components at *m* ≥ 2 would suggest a more complex dynamical interpretation, however, possibly associated with barotropic instability of the compact vortex core.

**Figure 13:**
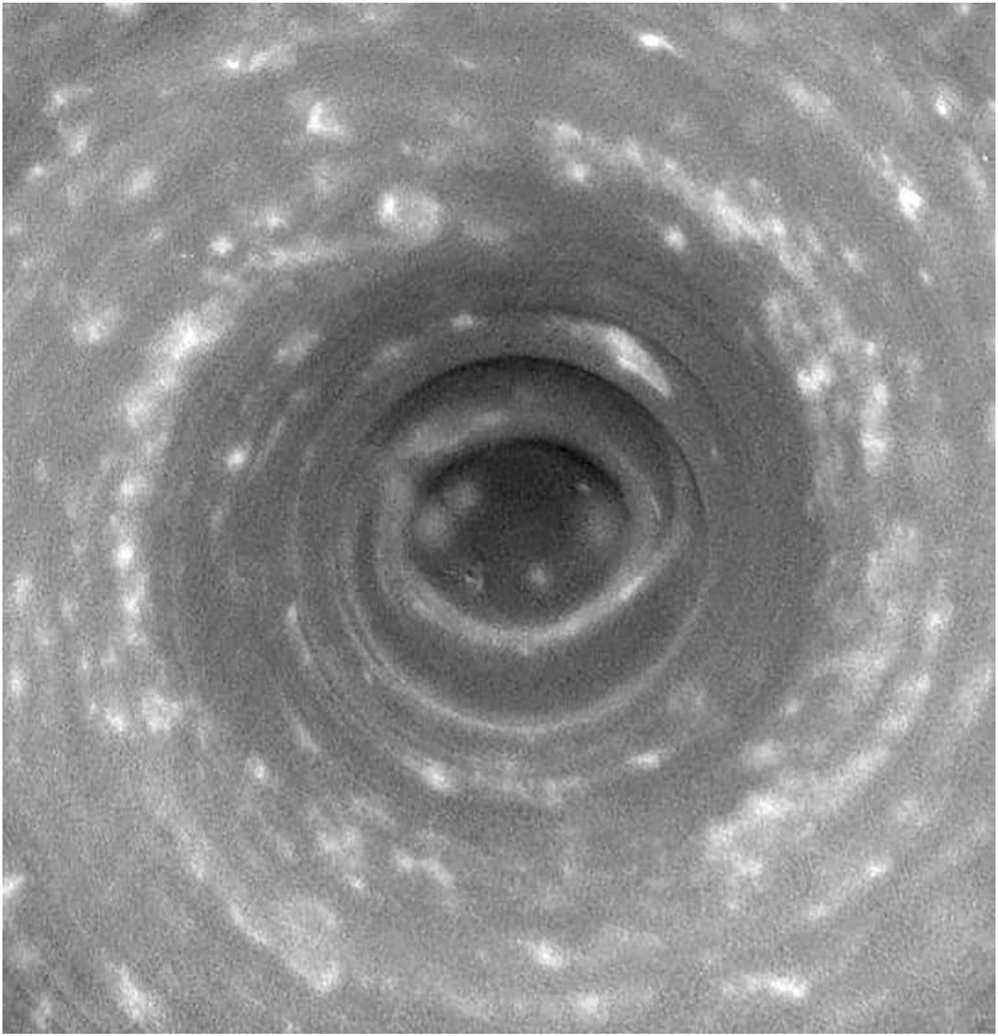
Image of the core of Saturn's South Polar Vortex, obtained by the Cassini Imaging Sub‐System wide‐angle camera using a spectral filter sensitive to wavelengths of infrared light centered at 752 nm on 11 October 2006. Image scale is about 17 km per pixel. Image credit from NASA/JPL/Space Science Institute, image no. PIA08332.

This contrasts with the SPV, where $\tilde{c\left(K_{E},K_{Z}\right)}\left(m,\phi \right)$ is distributed more broadly in latitude with systematic structure that is dominated by *m* ≥ 2 (especially *m* = 2 and *m* = 4 within 2° of the pole) without much of a contribution from *m* = 1 (see Figure 9d). Such a predominance of *m* = 2 is consistent with the elliptical appearance of the SPV in some images (e.g., see Figure 13).

The pattern of $\tilde{c\left(K_{E},K_{Z}\right)}\left(m,\phi \right)$ with latitude seems consistent with an acceleration of the axisymmetric vortex core within 2° of the pole from *m* = 2 and other even numbered harmonics, possibly suggestive of an acceleration of the vortex as an elliptical perturbation of the vortex decays. At lower latitudes the pattern is indicative of a tendency to flatten the outer zonal flow profile and displace a secondary peak in $\bar{u}$ at around 86°S equatorwards. Finally, $\tilde{c\left(K_{E},K_{Z}\right)}\left(m,\phi \right)$ in the SPJ (see Figure 9c) shows a systematic pattern of zonal flow acceleration from a wide range of zonal wavenumbers near the jet core, with weak deceleration on either side, mainly dominated by low wavenumbers *m* ≤ 10. This pattern indicates a similar trend to the NPJ, tending to sharpen the jet and strengthen its core, but with contributions spread across a wide range of *m* extending almost up to the resolution limit around *m* = 40.

**Figure 14:**
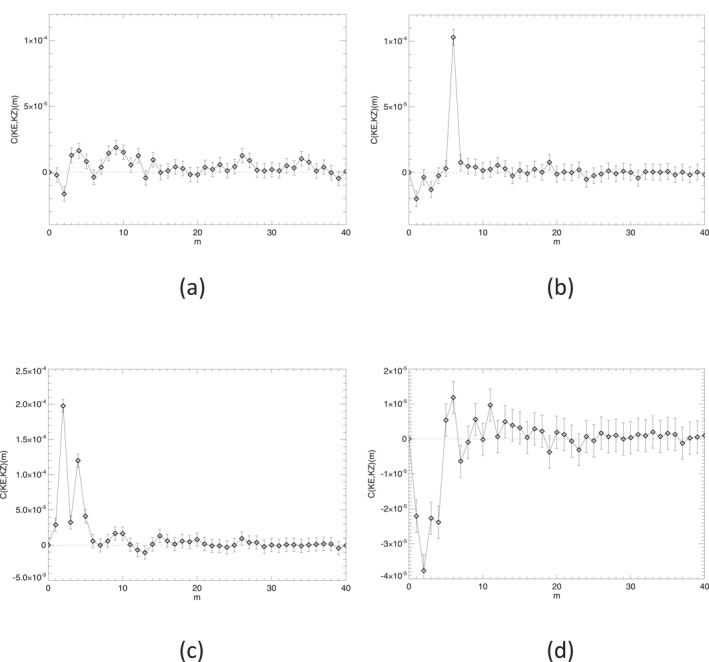
Spectrally resolved, eddy‐zonal flow kinetic energy conversion rate, $\tilde{C\left(KE,KZ\right)}\left(m\right)$ versus zonal wavenumber *m* for (a) Saturn's south polar jet (66°–76°S), (b) its north polar jet (70°–79°N), (c) the south polar vortex (80°–90°S) and (d) the north polar vortex (80°–90°N).

Integrating $\tilde{c\left(K_{E},K_{Z}\right)}\left(m,\phi \right)$ in latitude provides a determination of the overall contribution of each zonal wavenumber component to the generation of the KE of the zonal jet flow. Figure 14 shows results obtained from area‐weighted integrals of $\tilde{c\left(K_{E},K_{Z}\right)}\left(m,\phi \right)$ over the interval in latitude within ±5° of the NPJ and SPJ respectively. This shows the clear dominance of *m* = 6 in the north in transferring KE into the NPJ (Figure 14b) at a rate that is more than three times the mean conversion rate for the whole planet. $\tilde{C\left(K_{E},K_{Z}\right)}\left(m\right)$ is also positive for many other wavenumbers, though at a much lower level. Only *m* = 1, 3 and 4 seem to show a negative conversion rate in the NPJ region, indicating that they are gaining KE at the expense of the *m* = 0 zonal jet, although this might also reflect the impact of some large scale sampling errors. In the SPJ (Figure 14a), the contributions of individual wavenumber components are all relatively small in magnitude (<2–3 × 10^−5^ W kg^−1^ per wavenumber) though predominantly positive except at *m* = 1, 2, 4, 5 and 13. However, none of these components feature particularly strongly in the zonal KE spectrum for the southern polar region (cf. Figure 8a).

$\tilde{C\left(K_{E},K_{Z}\right)}\left(m\right)$ for the polar vortices shows a more complex and diverse situation between north and south. The SPV (Figure 14c) shows strong contributions to $\tilde{C\left(K_{E},K_{Z}\right)}\left(m\right)$ at *m* = 2 and *m* = 4, as remarked above, with only weak and probably insignificant contributions from other wavenumbers. For the NPV, however, Figure 14d suggests that low wavenumber structures (*m* ≤ 4) are drawing energy from the axisymmetric vortex while higher wavenumbers (*m* ≳ 4) are weakly feeding energy into the axisymmetric circumpolar jet surrounding the vortex, which error estimates suggest may be statistically significant unless measurement errors are heavily dominated by large scale sampling issues. This contrasting behavior between different wavenumber ranges may go some way to explaining the apparently large statistical error in *C*(*K*_*E*_, *K*_*Z*_) for the NPV (see Table 1).

## 5 Discussion

In this study we have analyzed the velocity fields in Saturn's polar regions, as derived by Antuñano et al. (2015), to evaluate the interactions between nonaxiymmetric eddies, waves and zonal jet flows. The results show that, with the exception of the vortices immediately encircling the poles, the overall tendency is for eddies to transfer KE into the zonal jets via horizontal Reynolds stresses at a rate that is similar to the rest of Saturn's atmosphere at latitudes equatorwards of 60° (Cabanes et al., 2020; Del Genio & Barbara, 2012; Del Genio et al., 2007). This tendency would therefore seem to be confirmed in the atmospheres of both Saturn and Jupiter, at least at the level of the cloud tops of both planets. The earlier analysis of Antuñano et al. (2015) was unable to reach a conclusion concerning the sense of KE transfers between eddies and the zonal mean jets in the vicinity of the NPJ and SPJ because of excessive noise and scatter in plots equivalent to Figure S5 in the Supporting Information S1. They only considered a rather narrower latitude band than was analyzed in Section 3.1 above, however, based on the raw, irregularly spaced velocity measurements. It may also be significant that their analysis defined *u*′ and *v*′ for the NPH as residuals following subtraction of a hexagonally meandering zonal jet rather than the conventional zonal mean $\bar{u}$ used here. In the present analysis, some smoothing in latitude was also applied to take account of the effective resolution of the image correlation algorithm, which also may have improved the signal‐to‐noise ratio of the measurements, especially in the zonal mean. As a result, the statistical analysis in Section 3.1 clearly demonstrated a statistically significant correlation consistent with a positive contribution to *C*(*K*_*E*_, *K*_*Z*_).

Perhaps the most striking result of the present analysis concerns the role of the NPH wave in the zonal KE budget. Through our zonal spectral decomposition, it seems quite clear that the *m* = 6 hexagon wave was directly transferring KE into the zonal mean NPJ at a rate approaching 200 μW kg^−1^. Unless this time period represents an unusual transient interval, therefore, when the NPH meanders happened to be decaying and giving up their KE to the zonal mean NPJ, this indicates that the NPH meanders were not being maintained as an active barotropic instability of the NPJ, at least at the time of the observations. If this were to be confirmed at other times, this would raise some significant questions that would need to be addressed by a whole class of explanations for the origin and maintenance of the NPH, including several recent numerical models and laboratory analogs (e.g., Aguiar et al., 2010; Farrell & Ioannou, 2017; Morales‐Juberías et al., 2015, 2011; Rostami et al., 2017). Our Figure 14b, for example, is directly comparable with Figure 4 of Farrell and Ioannou (2017) and shows the direct opposite of the *m* = 6 conversion rate obtained in their model. It is not clear whether our result is also inconsistent with the deep convection models of Yadav and Bloxham (2020) or Garcia et al. (2020) since they do not report on calculations of eddy‐zonal flow energetics in their papers, although the zonal jets produced in such models seem strongly barotropic in character. This would certainly be of interest to calculate in further modeling studies. A key goal for the future, however, should be to measure *C*(*K*_*E*_, *K*_*Z*_) for the NPH at other times to determine whether our results represent a transient phenomenon or the normal, equilibrated state of this feature of Saturn's atmosphere.

If our measurement does not represent a transient, however, then an alternative possibility that could be consistent with the results presented here is that an active baroclinic instability may be responsible for generating the *m* = 6 meanders in the NPJ. Several previous studies have shown that baroclinic instabilities can also develop into equilibrated polygonal meanders in a vertically sheared zonal jet (e.g., Bastin & Read, 1997, 1998; Hide & Mason, 1975; Morales‐Juberías et al., 2015; Sutyrin et al., 2001). In the presence of a *β*‐effect, this can lead to KE transfers from the eddies to the zonal flow, especially if the jet width is broader than the local baroclinic Rossby radius (Held & Andrews, 1983). Conclusive confirmation of this interpretation, however, would require explicit diagnosis of the baroclinic conversion rate from potential to eddy kinetic energy, involving both the large‐scale vertical velocity and temperature perturbations beneath the visible cloud tops. These are not available directly in observations, and may not be feasible to obtain for the foreseeable future. There is, however, some hint of a possible reversal of the northward PV gradient with altitude close to the NPJ around the level of the cloud tops at the time of these observations in the work of Antuñano et al. (2018) that might be suggestive of baroclinic processes. One of the model simulations of Morales‐Juberías et al. (2015) that reproduced a stable, hexagonal meandering jet in a shallow domain with vertical shear was also interpreted as a possible baroclinic instability, although this was not confirmed directly in other diagnostics.

The general tendency for $\tilde{C\left(K_{E},K_{Z}\right)}\left(m\right)$ to be positive for most values of *m* in both the NPJ and SPJ would seem to suggest that both jets could be weakly baroclinically unstable, allowing a statistically steady trickle of KE into their parent jets via conversion from available potential energy associated with horizontal temperature gradients around and below the visible cloud tops. If this was confirmed, it would suggest an analogy between both the NPJ and SPJ and the so‐called Ribbon Wave at 47° N on Saturn (e.g., Godfrey & Moore, 1986; Gunnarson et al., 2018; Sayanagi et al., 2010). The reason why the NPJ develops and maintains a strong *m* = 6 wave while the SPJ does not, however, remains somewhat mysterious and may require further observations and theoretical modeling, especially perhaps with regard to the structure of the flow beneath the visible cloud tops. Such a distinction has remained elusive to most models so far, including both shallow and deep convection scenarios.

As remarked previously, the polar vortices on Saturn are distinct structures with a closed, cyclonic circulation centered quite closely on each pole (Sánchez‐Lavega et al., 2006; Sayanagi et al., 2017). Images from Cassini have shown significant non‐axisymmetric perturbations to both vortices in the form of waves and smaller sub‐vortices (Baines et al., 2009; Dyudina et al., 2008, 2009; Sánchez‐Lavega et al., 2006). The SPV in particular was seen with an elliptical (*m* = 2) distortion in the eye wall (see Figure 13) while both the NPV and SPV exhibited spiral cloud features in their outer regions. The NPV also contained much smaller sub‐mesoscale vortices embedded within the spiral cloud bands indicating some complex local instabilities. It is noteworthy that our calculations of $\tilde{c\left(K_{E},K_{Z}\right)}\left(m,\phi \right)$ show a strong positive signal at *m* = 2 and 4 close to the south pole, consistent with the elliptical distortion of the vortex in the visible images. This would suggest that the elliptical perturbation to the vortex was actually contributing to strengthening the polar vortex itself close to its core, although further out from the core the contribution to $\tilde{C\left(K_{E},K_{Z}\right)}\left(m,\phi \right)$ seems consistent with *m* = 2 and 4 eddies weakly forcing a secondary jet at ∼86°S northwards. In the NPV, however, *m* = 2 appears to be making a weak negative contribution to $\tilde{c\left(K_{E},K_{Z}\right)}\left(m,\phi \right)$, suggestive of its tendency to grow at the expense of the axisymmetric vortex and consistent with a barotropic shear instability, although the contribution of *m* = 1 is positive. This should perhaps be examined more closely in future work.

Similarities between both polar vortices and terrestrial tropical cyclones have been noted previously for example, by Dyudina et al. (2009), who also point out the presence of many small anticyclones surrounding and embedded within Saturn's SPV. Tropical cyclones on Earth are often observed to develop non‐axisymmetric perturbations to their cores and eye walls (e.g., Kossin et al., 2002; Kossin & Schubert, 2001; Reasor et al., 2000; Schubert et al., 1999), mainly due to local transient barotropic shear instabilities, although they quickly break up and disperse on timescales of a few hours. Similar perturbations are seen in Venus's polar vortices (e.g., Limaye et al., 2009), which also show some resemblance to terrestrial tropical cyclone mesovortices. The perturbations to the Venus polar vortex appear also to be due to barotropic (and baroclinic?) shear instabilities (Limaye et al., 2009) which are strongly ageostrophic, much like in terrestrial cyclones where typical Rossby numbers *Ro* = *U*/*fL* ∼ *ζ*/*f* (where *ζ* is the local relative vorticity) are much greater than unity. For the Saturn polar vortices, *Ro* is typical O(1) (Antuñano et al., 2015; Dyudina et al., 2009; Sayanagi et al., 2017), suggesting planetary rotation may be somewhat more significant for their dynamical stability. As with other atmospheric features, their origin and depth of penetration into Saturn's deep interior remain highly uncertain (cf. Garcia et al., 2020). But our overall result that *C*(*K*_*E*_, *K*_*Z*_) ≲ 0 for the NPV (see Table 1) may be consistent with a weakly barotropically unstable vortex at the time of the Cassini measurements. It is likely that such instabilities are, like their terrestrial counterparts, dynamically active and transient, so it would be of significant interest, to analyze cloud motions around these features at other times to obtain more statistics on the occurrence and evolution of these unstable vortices.

Finally, we note that, given the high quality of images available from spacecraft such as Cassini, it would be desirable in future to take even fuller account of the potential sources of uncertainty in velocity measurements than has been possible in this study. In particular, our treatment of navigation errors here was relatively simple and straightforward, because foreshortening effects and other anisotropies were relatively small. But in general such errors may be strongly anisotropic and inhomogeneous across an image, for which the development of better methods may be desirable to quantify such uncertainties properly.

## Supporting information

Supporting Information S1

## Data Availability

Gridded velocity measurements from this study are available via the University of Oxford Research Archive (Antuñano et al., 2021) and Interactive Data Language (IDL) scripts to compute the diagnostics presented here and to plot the results are available via Zenodo (Read, 2022).
