# Supplementary material for: Energy Exchanges in Saturn's Polar Regions From Cassini Observations: Eddy‐Zonal Flow Interactions
Source: J Geophys Res Planets. 2022 Apr 27;127(5):e2021JE006973. doi: 10.1029/2021JE006973 (PMC9285433; doi:10.1029/2021JE006973)
Supplement: Supplementary file 1 — Supporting Information S1 [file JGRE-127-0-s001.pdf]

Supporting Information for “Energy exchanges in Saturn’s polar regions from Cassini observations: Eddy-zonal flow interactions”

Peter L. Read[1], Arrate Antuñano[2], Simon Cabanes[3], Greg Colyer[1],

Teresa del Río Gaztelurrutia[2], and Agustin Sanchez-Lavega[2]

[1]Atmospheric, Oceanic and Planetary Physics, Department of Physics, University of Oxford, Clarendon Laboratory, Parks Road, Oxford, OX1 3PU, UK

[2]Dpto de Física Aplicada, Escuela de Ingeniería de Bilbao, UPV/EHU, Spain

[3]DICEA, Sapienza Università di Roma, Via Eudossiana, 18 - 00184, Rome, Italy

Contents of this file

1. Commentary on additional figures
2. Figures S1 to S7

In the following section we include additional figures that supplement the information provided in the main text. Figure S1 shows the distribution in latitude and longitude of the original velocity vectors as measured by Antuñano et al. (2015). Figure S2 shows the distribution of interpolated points on regular longitude-latitude grids for each of the northern and southern hemispheres. Figure S3 presents maps of u and v for the southern hemisphere with a segment of data from longitudes 110° - 185° inserted into the data

gap between 35° and 110° longitude, as an example of how this gap was filled to enable Fourier analyses of the zonal structure of the flow.

Figure S4 presents profiles in latitude of the (Pearson) correlation coefficients between u' and v' for the northern and southern hemispheres. For this calculation, data were analysed as the running mean in latitude over a 1° interval to reflect the effective resolution of the original velocity measurements.

Figure S5 shows the pointwise distribution of $\overline{u'v'}$ vs $d\overline{u}/dy = (1/a)\cos\phi d(\overline{u}/\cos\phi)/d\phi$ for (a) the vicinity of the NPJ and (b) the SPJ (±4° of the jet cores). Athough there is quite a lot of scatter among the points in both jets, even by eye there is some indication of a positive correlation between $\overline{u'v'}$ and $\partial\overline{u}/\partial y$ in both frames, consistent with a positive contribution to $C(K_E, K_Z)$. Calculation of the Pearson correlation coefficients for each of the NPJ and SPJ latitude bands with 1° latitude smoothing gives values of 0.59 and 0.40, whereas the Spearman (non-parametric) rank correlation coefficients from these data yields values of 0.47 and 0.54 respectively, indicating a rejection of the null (uncorrelated) hypothesis at the $> 99\%$ confidence level.

Figure S6 presents an alternative version of Figure 9 in the main text but using linear scales for both wavenumber and spectral energy density.

Figure S7 compares the observed structure of u and v (frames (a) and (c)) with the flow corresponding only to the $m = 0$ and $m = 6$ zonal Fourier components ((b) and (d)).

References

Antuñano, A., del Río-Gaztelurrutia, T., Sánchez-Lavega, A., & Hueso, R. (2015). Dynamics of Saturn's polar regions. *J. Geophys. Res.: Planets*, *120*, 155–176. doi: 10.1002/2014JE004709

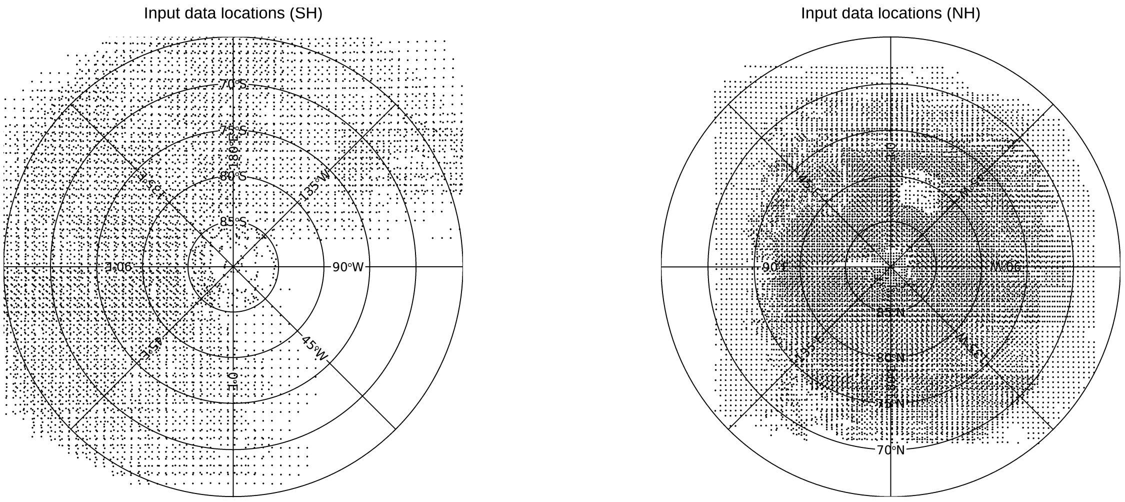

Figure S1. Locations in longitude and latitude of the original velocity vector measurements as obtained by Antuñano et al. (2015). Note that velocity vectors were not available in the south equatorwards of 76°S between longitudes of ∼ 35°-110°W.

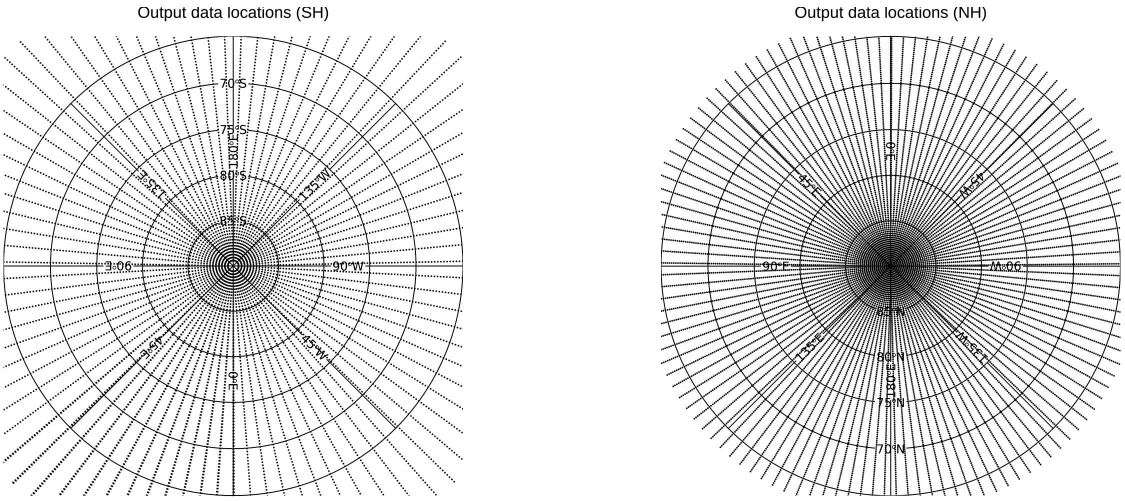

Figure S2. Locations in longitude and latitude of the velocity vector measurements as obtained by Antuñano et al. (2015) interpolated onto regular longitude-latitude grids for each hemisphere.

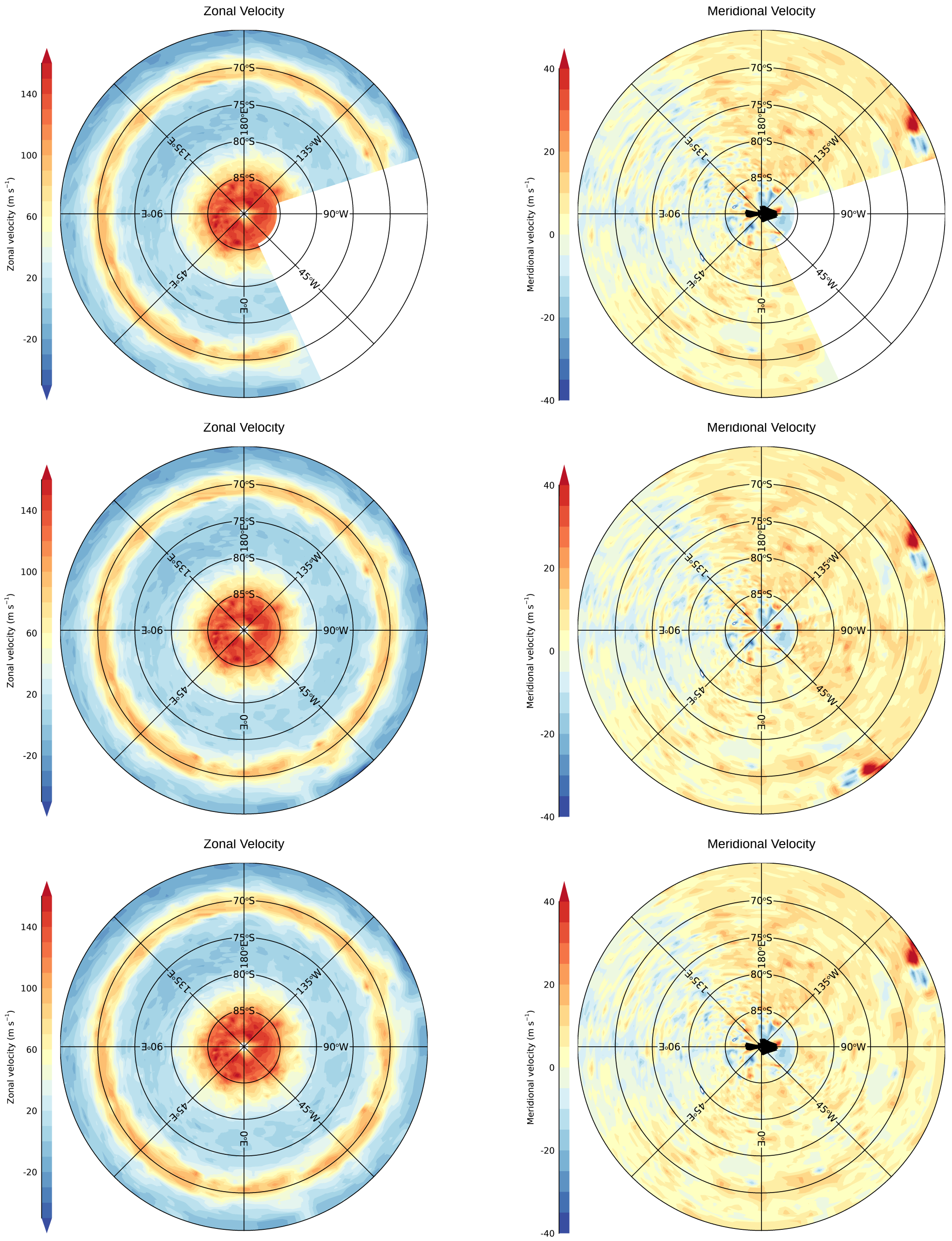

Figure S3. Cloud-top level velocity fields, obtained from cloud-tracked wind measurements using Cassini ISS images by Antuñano et al. (2015), for Saturn's (a) south polar region. As discussed in the main text, velocity vectors were not available in the south equatorwards of 76°S between longitudes of ∼ 35°-110°W, as indicated in (a) and (b). The fields shown in (c) and (d), and (e) and (f) were obtained by inserting data from longitudes 110°-185° [(c) and (d)] or 320°-35° into the data gap, to illustrate two examples of how the data gap was filled before carrying out zonal Fourier analysis.

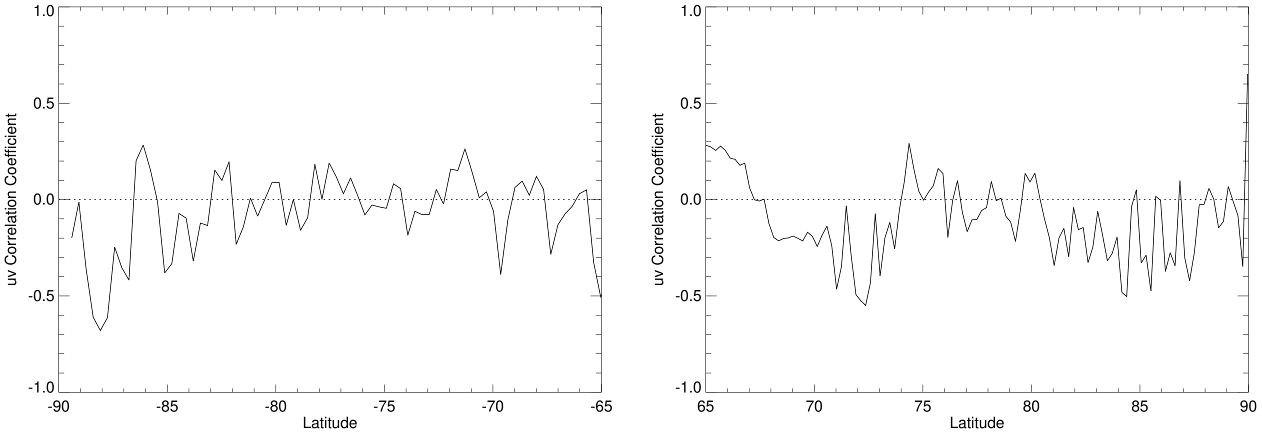

Figure S4. Profiles in latitude of the Pearson correlation coefficient between u' and v' in longitude for (a) the southern and (b) the northern hemispheres of Saturn from the data of Antuñano et al. (2015). Interpolated data were smoothed in latitude using a running mean of width 1° before computing the correlation coefficient (in longitude).

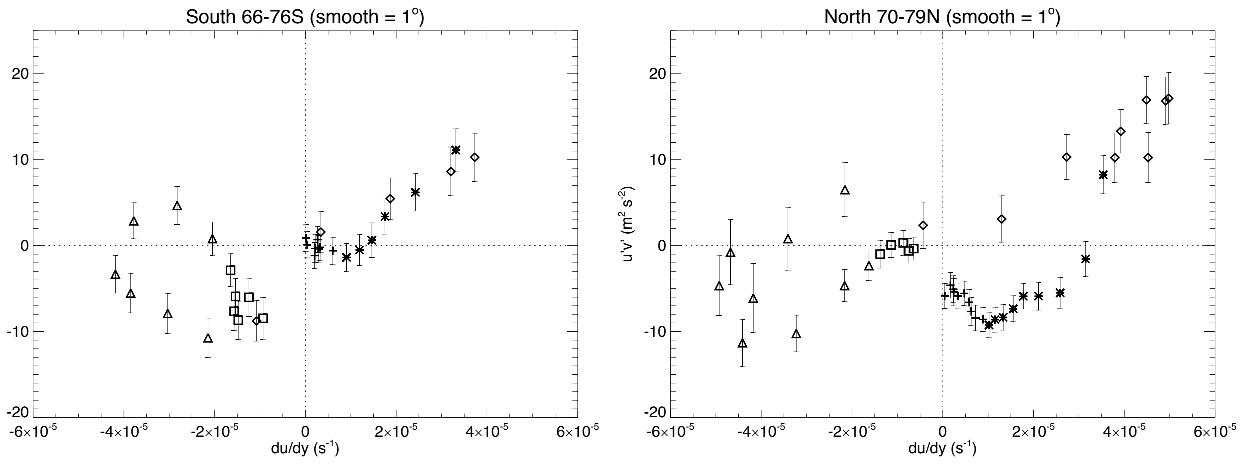

(a) (b)

Figure S5. Pointwise scatter plots of eddy momentum flux, $\overline{u'v'}$, vs $\partial\overline{u}/\partial y$ for (a) Saturn's south polar jet region (66°-76° S) and (b) the south polar jet region (72°-80° N. Both plots use a smoothing in latitude of width $\sim 1°$. Symbols plotted indicate the latitude band from which each point is taken. For (a) squares are from 66°-68°S, triangles from 68°-70°S, diamonds from 70°-72°S, stars from 72°-74°S and crosses are from 74°-76°S. For (b), squares are from 78°-79°N, triangles from 76°-78°N, diamonds from 74°-76°N, stars from 72°-74°N and crosses are from 70°-72°N.

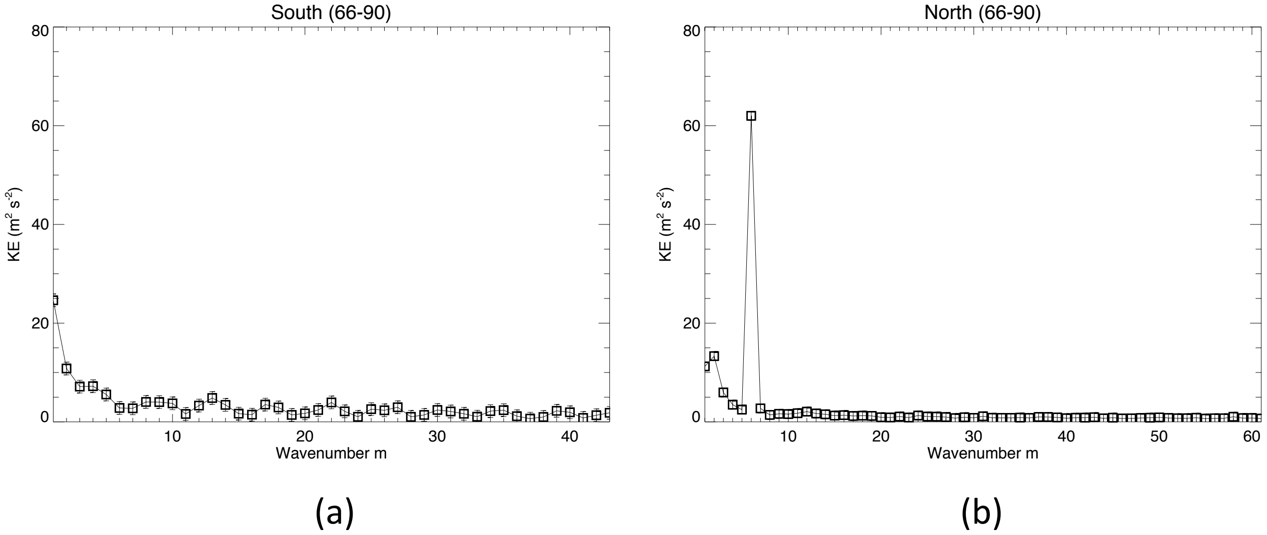

Figure S6. Area-weighted kinetic energy spectra for (a) the southern and (b) the northern polar regions (66°–90° latitude).

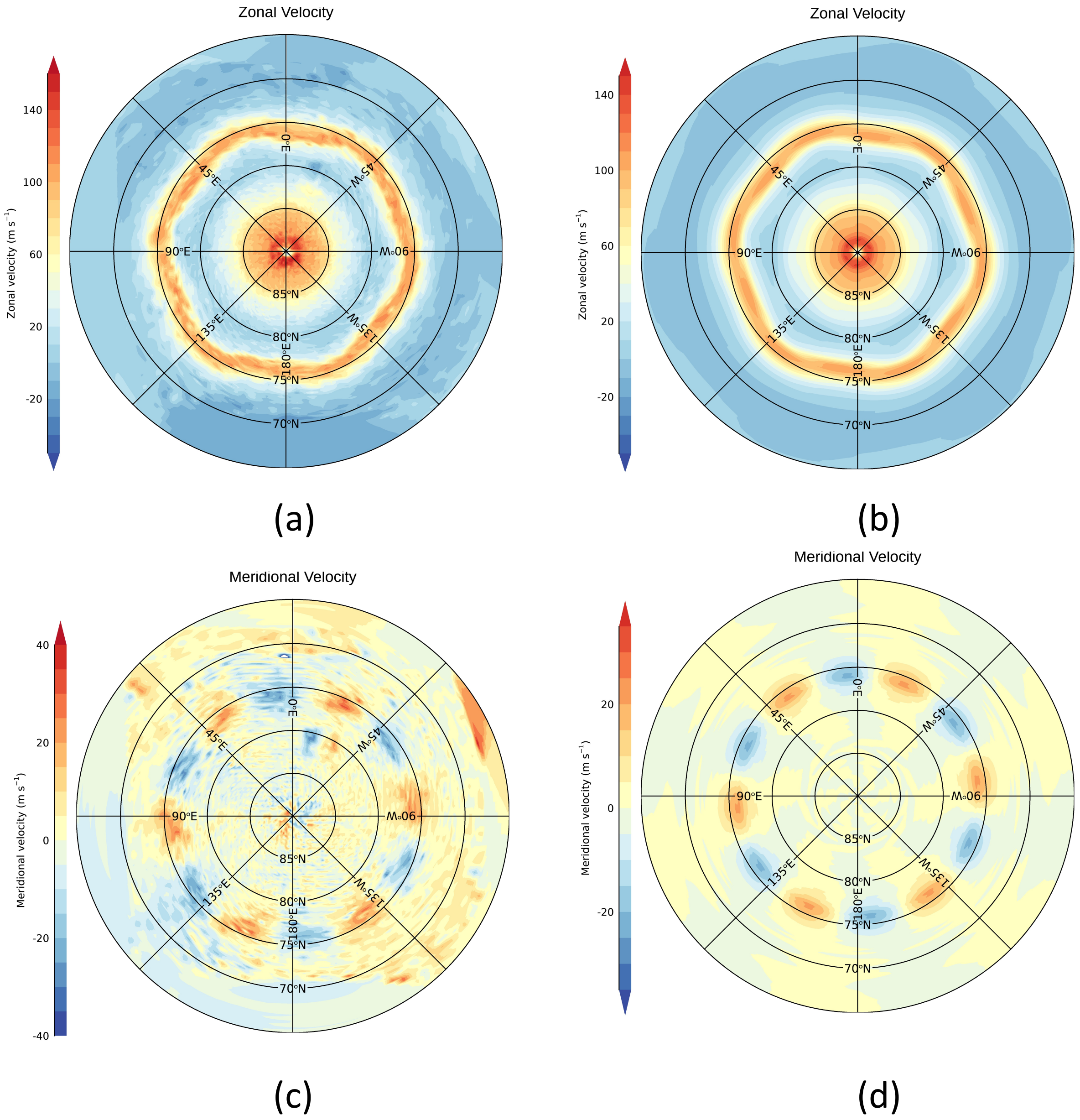

Figure S7. Comparison between the complete u and v components of the velocity field in the northern polar latitudes of Saturn ((a) and (c)), as obtained by Antuñano et al. (2015), and the contributions solely due to the $m = 0$ and $m = 6$ zonal Fourier harmonics ((b) and (d)). In the vicinity of the NPH these two harmonics capture more than 93% of the total kinetic energy.
